# Keeping things local: Subpopulation *N*_b_ and *N*_e_ in a stream network with partial barriers to fish migration

**DOI:** 10.1111/eva.12454

**Published:** 2017-02-09

**Authors:** Andrew R. Whiteley, Jason A. Coombs, Matthew J. O'Donnell, Keith H. Nislow, Benjamin H. Letcher

**Affiliations:** ^1^ Wildlife Biology ProgramDepartment of Ecosystem and Conservation SciencesCollege of Forestry and ConservationUniversity of MontanaMissoulaMTUSA; ^2^ Department of Environmental ConservationUniversity of Massachusetts AmherstAmherstMAUSA; ^3^ U.S. Forest ServiceNorthern Research StationUniversity of MassachusettsAmherstMAUSA; ^4^ U.S. Geological SurveyLeetown Science CenterS.O. Conte Anadromous Fish Research CenterTurners FallsMAUSA

**Keywords:** brook trout, effective number of breeders, effective population size, linkage disequilibrium, metapopulation, salmonid, stream fishes, temporal estimator

## Abstract

For organisms with overlapping generations that occur in metapopulations, uncertainty remains regarding the spatiotemporal scale of inference of estimates of the effective number of breeders (${\hat{N}}_{b}$) and whether these estimates can be used to predict generational *N*_e_. We conducted a series of tests of the spatiotemporal scale of inference of estimates of *N*_b_ in nine consecutive cohorts within a long‐term study of brook trout (*Salvelinus fontinalis*). We also tested a recently developed approach to estimate generational *N*_e_ from ${\hat{N}}_{b}$ and compared this to an alternative approach for estimating ${\hat{N}}_{e}$ that also accounts for age structure. Multiple lines of evidence were consistent with ${\hat{N}}_{b}$ corresponding to the local (subpopulation) spatial scale and the cohort‐specific temporal scale. We found that at least four consecutive cohort‐specific estimates of ${\hat{N}}_{b}$ were necessary to obtain reliable estimates of harmonic mean ${\hat{N}}_{b}$ for a subpopulation. Generational ${\hat{N}}_{e}$ derived from cohort‐specific ${\hat{N}}_{b}$ was within 7%–50% of an alternative approach to obtain ${\hat{N}}_{e}$, suggesting some population specificity for concordance between approaches. Our results regarding the spatiotemporal scale of inference for *N*_b_ should apply broadly to many taxa that exhibit overlapping generations and metapopulation structure and point to promising avenues for using cohort‐specific ${\hat{N}}_{b}$ for local‐scale genetic monitoring.

## 1 Introduction

Effective population size (*N*_e_) is defined as the size of a theoretical and ideal population affected by genetic drift at the same rate per generation as the population under consideration (Wright, 1931). *N*_e_ directly determines the rate of genetic drift and captures information about the relative importance of natural selection, mutation, and migration (Hare et al., 2011; Luikart, Ryman, Tallmon, Schwartz, & Allendorf, 2010; Waples, 2005, 2010). Recent advances in methods used to estimate contemporary *N*_e_ (Jorde & Ryman, 2007; Pudovkin, Zaykin, & Hedgecock, 1996; Tallmon, Koyuk, Luikart, & Beaumont, 2008; Wang, 2009; Wang & Whitlock, 2003; Waples, 1989; Waples & Do, 2008) have led to a dramatic increase in studies that have estimated *N*_e_ in natural populations (Palstra & Fraser, 2012; Waples, 2010). However, uncertainties related to estimation of *N*_e_ in iteroparous organisms remain.

For iteroparous organisms, it is challenging to reliably estimate the effective size of an entire generation, or generational *N*_e_ (Waples, Antao, & Luikart, 2014; Waples & Do, 2010; Waples & Yokota, 2007). An alternative approach for organisms with this life history is to apply single‐sample *N*_e_ estimators to single cohorts (defined as a group of individuals born in a given year and thus with the same age; Caswell, 2001) to obtain an estimate of the effective number of breeders (*N*_b_) that gave rise to each cohort (Palstra & Fraser, 2012; Waples, 2005; Waples et al., 2014; Whiteley et al., 2012, 2015). Recent works suggest that single cohort *N*_b_ provides a metric of population‐specific individual reproductive contribution and therefore could form an important component of genetic monitoring programs (Whiteley et al., 2015). However, challenges to interpreting single‐cohort *N*_b_ remain; in particular, the appropriate scale of spatial or temporal inference for estimates of ${\hat{N}}_{b}$ is often not clear.

Connected sets of subpopulations with ongoing gene flow provide a considerable challenge for interpreting the spatial scale of inference for estimates of ${\hat{N}}_{b}$ (Gomez‐Uchida, Palstra, Knight, & Ruzzante, 2013; Serbezov, Jorde, Bernatchez, Olsen, & Vollestad, 2012b; Wang & Whitlock, 2003; Waples, 2010; Waples & England, 2011). Gene flow could increase the spatial scale beyond local (subpopulation) ${\hat{N}}_{b}$ (Waples & England, 2011). As migrants enter a subpopulation, a larger number of parents could contribute to the next cohort, which could expand the spatial scale to which estimators of ${\hat{N}}_{b}$ apply beyond the local subpopulation (Waples & England, 2011). Alternatively, for the most commonly used linkage disequilibrium (LD)‐based estimator, genetically divergent immigrants could create LD due to population mixture (so called “mixture LD”) (Neel et al., 2013; Palstra & Ruzzante, 2011; Waples & England, 2011; Waples et al., 2014) or admixture (Nei & Li, 1973; Sinnock, 1975) that could downwardly bias estimates of local *N*_b_ (Park, 2011; Waples et al., 2014). Several studies have used simulations to test these effects (Neel et al., 2013; Waples & England, 2011). For example, island model simulations found that LD‐based estimates of *N*_e_ reflect local subpopulation *N*_e_ unless equilibrium gene flow (*m*) > 5%–10%. Larger *m* led to convergence to global (metapopulation) *N*_e_ under some conditions (Waples & England, 2011). Empirical data from thoroughly examined population systems would complement these simulation studies and help define spatial effects on *N*_b_ in connected sets of populations.

In addition to spatial uncertainty regarding ${\hat{N}}_{b}$ inferences, it is also unclear whether the temporal inference for ${\hat{N}}_{b}$ corresponds to a single cohort or if there are “legacy” genetic effects. Single‐cohort *N*_b_ estimates apply to a combination of (i) the single reproductive event that gave rise to a cohort and (ii) legacy genetic effects that persist over the past one or few generations (Waples, 1991). The LD signal used by LD estimators results from few parents that generate either few families, high variance in family sizes, or both (Luikart et al., 2010; Waples, 1991, 2010; Whiteley et al., 2015). As LD for unlinked loci decays by 50% per generation, estimators based on unlinked loci should be primarily sensitive to *N*_e_ of the parental generation (assuming discrete generations) or *N*_b_ of the parents of the cohort of interest (assuming overlapping generations and single‐cohort sampling) (Waples, 2005, 2010; Whiteley et al., 2015), but this has received little attention to date.

While ${\hat{N}}_{b}$ appears to be more estimable and interpretable for organisms with age structure, generational ${\hat{N}}_{e}$ is useful because it provides a direct link to a rich body of population genetic theory. A new approach provides a way to use ${\hat{N}}_{b}$ to estimate generational ${\hat{N}}_{e}$ (Waples et al., 2014). This new approach requires a few key life history traits (age at maturity and adult life span) or full life tables (Waples, Luikart, Faulkner, & Tallmon, 2013; Waples et al., 2014). Empirical evaluations would help to define possible limitations of this new approach. While some past research has compared ${\hat{N}}_{b}$ to ${\hat{N}}_{e}$ within study systems (Charlier, Laikre, & Ryman, 2012; Palstra, O’Connell, & Ruzzante, 2009), there have been few empirical attempts to compare ${\hat{N}}_{e}$ from the new Waples et al. (2014) approach to empirical estimates of generational ${\hat{N}}_{e}$ (but see Ruzzante et al., 2016). This evaluation requires robust estimates of harmonic mean ${\hat{N}}_{b}$ and ${\hat{N}}_{e}$ from the same study system, which are best obtained from multiple cohorts from the same population (Jorde & Ryman, 1995). The number of cohorts needed to obtain reliable estimates of harmonic mean ${\hat{N}}_{b}$ remains unclear, as cohort‐specific ${\hat{N}}_{b}$ is likely to fluctuate over time due to environmental effects and variation in age at maturity (Whiteley et al., 2015).

Studies of stream fishes are well suited for gaining a comprehensive understanding of a species’ population biology, genetic structure, and effective population size within interconnected population systems. The linear nature of stream habitats simplifies analyses of population genetic structure (Kanno, Vokoun, & Letcher, 2011; Koizumi, Yamamoto, & Maekawa, 2006; Serbezov, Jorde, Bernatchez, Olsen, & Vollestad, 2012a; Whiteley et al., 2010). Extensive sampling can be used to obtain robust estimates of effective size and abundance from multiple consecutive cohorts (Whiteley et al., 2015; Wood, Belmar‐Lucero, Hutchings, & Fraser, 2014). It is also possible to narrow the focus on key environmental variables that might influence measures of effective population size. For example, work with salmonids has revealed that stream flow and temperature are important environmental factors that can be used to help predict species’ distributions under climate change (Isaak, Young, Nagel, Horan, & Groce, 2015; Wenger et al., 2011). Other work has demonstrated strong relationships between stream flow, temperature, and demographic rates (Bassar, Letcher, Nislow, & Whiteley, 2016; Letcher et al., 2014).

Here, we estimate ${\hat{N}}_{b}$, ${\hat{N}}_{e}$, and ${\hat{N}}_{C}$ (defined as the abundance of reproductively mature individuals) in a long‐term study of a stream‐dwelling salmonid, the brook trout (*Salvelinus fontinalis*). Our first objective was to examine the spatial and temporal scale to which estimates of ${\hat{N}}_{b}$ apply within this stream network. First, we defined population substructure, including estimates of admixture. We then examined single‐cohort estimates of ${\hat{N}}_{b}$ across nine cohorts within subpopulations. We conducted a series of five tests of whether ${\hat{N}}_{b}$ from subpopulations within a metapopulation apply to a local (subpopulation) scale and a cohort‐specific timescale. Our second objective was to compare two empirical estimates of ${\hat{N}}_{e}$ that account for age structure in different manners. We used ${\hat{N}}_{b}$ to predict generational ${\hat{N}}_{e}$ based on the Waples et al. (2014) approach and to compare ${\hat{N}}_{e}$ obtained from cohort‐specific ${\hat{N}}_{b}$ to robust empirical estimates of ${\hat{N}}_{e}$ obtained with the Jorde and Ryman (1995) approach. This work helps to establish ${\hat{N}}_{b}$ as a genetic metric that can be used for cohort‐ and subpopulation‐specific genetic monitoring and helps define the data requirements for obtaining estimates of generational ${\hat{N}}_{e}$ from ${\hat{N}}_{b}$.

## 2 Methods

### 2.1 Study site

**Figure 1:**
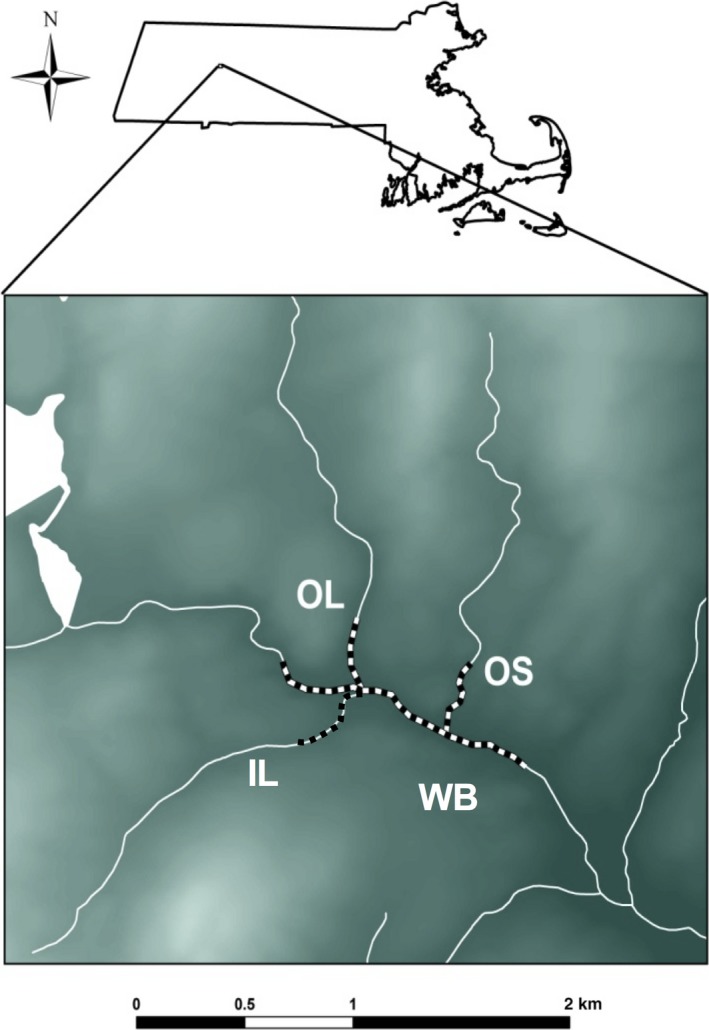
Map of study area of the West Brook, Massachusetts, USA. Brook trout were sampled four times per year from the black and white highlighted area. Natural waterfalls that serve as barriers to fish movement occur at the upstream extent of the highlighted portions of OS, OL, and IL, and at the mouth of IL. The tributaries empty into WB and the direction of stream flow is from left to right for WB. PIT tag antennas are located at the upper end of the study reach (WB) and the mouth of tributaries (OS, OL, IL)

Our study site is the West Brook (hereafter WB) and three tributary streams located in Western Massachusetts, USA, and described in detail by Kanno, Letcher, Coombs, Nislow, and Whiteley (2014), Letcher, Nislow, Coombs, O'Donnell, and Dubreuil (2007) and Letcher et al. (2014) (Figure 1). We have previously examined environmental influences of population demography (Bassar et al., 2016; Letcher et al., 2014), connectivity (Kanno et al., 2014), and single‐cohort ${\hat{N}}_{b}$ at the metapopulation scale in this long‐term study (Whiteley et al., 2015). The focal study area consists of a 1‐km‐long reach of the mainstem WB and 0.3‐km‐long reaches of three tributaries (Open‐Large, OL; Open‐Small, OS; Isolated‐Large, IL). The lower (or downstream) end (or boundary) of the study area of the WB contains a small but passable waterfall. The upper (or upstream) end (or boundary) of the WB study area is unobstructed. A waterfall (2.3 m) prevents access to IL from the WB. Large (>4 m, OL, OS) or small (1 m, IL) waterfalls delimit the upstream ends of the tributary study reaches. Mean stream width is 4.5 m for the WB, 3 m for OL, 2 m for IL, and 1 m for OS.

Naturally reproducing populations of brook trout occur in all four streams within this stream network. Fish regularly move among WB, OS, and OL (Kanno et al., 2014) but should not be able to enter IL (Letcher et al., 2007). A perched culvert limited entry into OS under low flow conditions (Kanno et al., 2014). A strong shift toward the demographic importance of smaller fish has been observed in IL relative WB, OS, and OL (Letcher et al., 2007).

Fish were sampled in a spatially continuous manner on four occasions (spring = late March, summer = June, autumn = late September and early October, winter = early December) per year from 2001 to 2009 using double‐pass electrofishing and block nets at the ends of 20‐m sections (Kanno et al., 2014). The WB has been sampled since 2001, and sampling of the tributaries began in 2002. Upon capture, fish were measured, weighed, and tagged with passive integrated transponder (PIT) tags, and an anal fin clip was taken as a tissue sample. Fish were assigned to cohorts based on length frequency histograms and a growth model (Letcher et al., 2014). We used a subset of the demographic data reported in Letcher et al. (2014) here, which consisted of a total of 20,089 observations of 10,458 individual trout.

### 2.2 Genetic analyses

We examined variation at 12 microsatellite loci in 6,932 fish from the 2001 through 2009 cohorts. DNA was extracted from fin clip tissue samples following a standard salt precipitation procedure. We used the following loci: *Sfo*‐*C113*,* Sfo*‐*C88*,* Sfo*‐*D75*,* Sfo*‐*D100*,* Sfo*‐*C24*,* Sfo*‐*C115*,* Sfo*‐*C129* (King, Julian, Coleman, & Burnham‐Curtis, 2003), *Ssa*‐*D237* (King, Eackles, & Letcher, 2005), *Sfo*‐*C38*,* Sfo*‐*C86*,* Sfo*‐*B52*, and *Sfo*‐*D91a* (King, Lubinski, Burnham‐Curtis, Stott, & Morgan, 2012). DNA extraction and amplification followed Whiteley et al. (2013). Loci were electrophoresed on an ABI Prism 3130xl genetic analyzer (Applied Biosystems Inc., Foster City, CA, USA), and alleles were hand, scored using GENEMAPPER version 3.2 and PEAK SCANNER version 1.0 (Applied Biosystems Inc.). Positive controls of brook trout with known genotypes were used for each set of PCR and electrophoresis. We previously reported a per‐allele genotyping error rate of 0.32% for this data set (Whiteley et al., 2015).

### 2.3 Tests of genetic structure

We needed to define genetic population structure within the stream network prior to analysis of *N*_b_ and *N*_e_. Previous analyses based on tagging and sibship reconstruction of a subset of the data presented here revealed high rates of movement among WB, OS, and OL (Kanno et al., 2014; Whiteley et al., 2015). We used STRUCTURE ver. 2.3.1 (Pritchard, Stephens, & Donnelly, 2000) to define genetic structure. A study of riverine brook trout found evidence that age‐0 fish (YOY) are captured near their natal locations (Hudy, Coombs, Nislow, & Letcher, 2010). We therefore performed an initial STRUCTURE analysis with YOY‐only, to maximize the probability that an individual was sampled in its stream of birth. To minimize bias associated with family‐level (sibling) effects (Allendorf & Phelps, 1981; Anderson & Dunham, 2008; Rodriguez‐Ramilo & Wang, 2012), we performed this STRUCTURE analysis with one randomly sampled full‐sibling per family. Prior to STRUCTURE analyses, we reconstructed full‐sibling families for all individuals assigned to each cohort with COLONY v 1.2 (Wang, 2004) following Whiteley et al. (2015). A previous study based on empirically parameterized simulations confirmed high accuracies of sibship reconstruction in WB based on the same 12 microsatellite loci using this version of COLONY (Letcher, Coombs, & Nislow, 2011). Reconstructed full‐sib families composed of at least two individuals had a rate of correct family inference of 91.2% (0.7% SE). For full‐sib families composed of at least five individuals, the rate of correct family inference was 97.7% (0.4% SE) (Letcher et al., 2011).

For the initial YOY‐only STRUCTURE analysis, we used 250,000 replicates and 25,000 burn‐in cycles under an admixture model for each STRUCTURE run with no location prior. We inferred a separate Dirichlet parameter for degree of admixture (α) for each subpopulation. We used the correlated allele frequencies model with an initial λ of 1, where λ parameterizes the allele frequency prior and is based on a Dirichlet distribution of allele frequencies. We allowed *F* to assume a different value for each subpopulation, which allows for different rates of drift among subpopulations. We evaluated LnP(*D*) for five replicates of *K *=* *1–10. We also used the Evanno approach (Evanno, Regnaut, & Goudet, 2005) to help evaluate *K* for each of the 10 replicates. We used CLUMPP ver. 1.1.2 (Jakobsson & Rosenberg, 2007) to obtain average *q*‐values across the five replicates of each of the 10 subsamples. Individuals were assigned to natal subpopulations if *q *>* *.70 and were considered admixed if .30 < *q *<* *.70. We repeated these procedures for 10 separate randomly subsampled (full‐sibling‐purged) data sets to test for an effect of our full‐sib subsampling approach on inference of the number of genetic clusters (*K*) with STRUCTURE.

After observing little effect of subsampling on the inference of *K*, we randomly chose one of the 10 STRUCTURE replicates to evaluate deviations from HW proportions and LD. The markers used here have not exhibited HW violations or LD in mixed‐aged samples in other populations examined (Annett, Gerlach, King, & Whiteley, 2012; Kanno et al., 2011; Kazyak et al., 2015). We reported extensive testing of Hardy–Weinberg (HW) proportions and linkage (gametic) disequilibrium (LD) previously for this data set (Whiteley et al., 2015). We previously concluded that family structure (number and size of family groups) and population substructure within the connected sites (WB, OS, and OL) were most likely responsible for the signal of HW deviations and LD (Whiteley et al., 2015).

We performed a second STRUCTURE analysis with all individuals from each cohort to assign each individual to its putative stream of origin and subsequently to allow us to estimate cohort‐specific ${\hat{N}}_{b}$ for each subpopulation. We performed only replicates of the admixture model with *K *=* *3 (the most supported model based on YOY‐only and one full‐sib per family across the 10 replicates; see Results) and no location prior. Family structure was present in this analysis, but the purpose of the analysis was not inference of *K*, rather the purpose was to obtain *q*‐values for all individuals genotyped.

All subsequent analyses were performed on the entire data set after subpopulations were defined and all putative migrants (*q *>* *.70) were assigned to their putative subpopulation of origin. We calculated summary statistics for each cohort within each subpopulation. We used CREATE version 1.33 (Coombs, Letcher, & Nislow, 2008) to make input files for FSTAT version 2.9.3.2 (Goudet, 1995, 2001) and GENEPOP version 4.0.10 (Rousset, 2008). We calculated mean number of alleles per cohort (*A*_O_); mean allelic richness, standardized to the cohort with the lowest number of individuals (*A*_R_); mean expected heterozygosity (*H*_S_); and *F*_IS_ for each cohort.

We also examined patterns of genetic differentiation over time within and among subpopulations. We used Nei's unbiased estimator of *G*_ST_ (Nei, 1987) as a measure of pairwise *F*_ST_ and Meirmans and Hedrick's unbiased estimator $G_{\mathrm{ST}}^{\prime \prime }$ (Meirmans & Hedrick, 2011) as a measure of pairwise $F_{\mathrm{ST}}^{\prime }$ among WB/OS, OL, and IL (based on *K *=* *3 from STRUCTURE). Both *F*_ST_ and $F_{\mathrm{ST}}^{\prime }$ were calculated with GENODIVE version 2.0b27 (Meirmans & Van Tienderen, 2004). We combined locus‐specific exact tests for allele frequency (genic) differentiation implemented in GENEPOP with Fisher's method. This test assumes that, under the null hypothesis of no allele frequency differentiation at any of the 12 loci, the quantity $-2\sum \text{ln}p_{j}$ is distributed as a χ^2^ with *df* = 2*k*, where *k* is the number of loci and *p*_j_ is the *p* value for the *j*th locus (Ryman et al., 2006). We compared the three subpopulations within each cohort (3 subpopulations × 9 cohorts = 27 pairwise comparisons). We used a sequential Bonferroni correction method (Rice, 1989) to control the type I error rate (α = .05) for results from this combined test. We used principal component analysis (PCA) of cohort‐specific allele frequencies to visualize patterns of population differentiation. We performed eigenanalysis based on a covariance with GENODIVE. We used the unbiased estimator $F_{S}^{\prime }$ (*F*_S_ corrected for sample size) as a measure of temporal allele frequency variation among cohorts (Jorde & Ryman, 2007).

### 2.4 ${\hat{N}}_{b}$ and ${\hat{N}}_{C}$ over time within subpopulations

We used a new beta version of the program LDNe (Waples & Do, 2008) (R. Waples pers. comm.) to obtain estimates of ${\hat{N}}_{b}\left({\hat{N}}_{b-\mathrm{LDNe}}\right)$ for the STRUCTURE‐defined subpopulations and for each cohort. LDNe provides the most extensively tested single‐sample effective population size estimator (Luikart et al., 2010). Estimates were calculated assuming random mating and excluding alleles with frequencies <.02. *P*_crit_ = .02 has been shown to provide an adequate balance between precision and bias across sample sizes (Waples & Do, 2008). Bias can be most severe when singleton alleles are included in a population sample (alleles that occur at a frequency of 1/2*S*, where *S *= sample size) (Waples & Do, 2008). In our case, the smallest sample size occurred in IL in the 2006 cohort (*S *=* *53). Singleton alleles were filtered from this and all other cohort samples with *P*_crit_ = .02. The beta version of LDNe implements a corrected version of a new jackknife approach over individuals (Jones, Ovenden, & Wang, 2016) while producing identical point estimates as previous versions. Other single‐sample estimators of ${\hat{N}}_{b}$ are available (Tallmon et al., 2008; Wang, 2009) but were biased low when applied to simulated brook trout data (A. Whiteley, unpublished results).

We compared ${\hat{N}}_{b}$ to abundance estimates (${\hat{N}}_{C}$) for each subpopulation. Abundance estimates followed the approach from Whiteley et al. (2015) and are based on data used for detailed demographic analyses (Bassar et al., 2016; Letcher et al., 2014). We define ${\hat{N}}_{C}$ as the count of age‐1 and older fish divided by the probability of capture (*p*). The appropriate ${\hat{N}}_{C}$ for comparison to ${\hat{N}}_{b-\mathrm{LDNe}}$ from year *t* was from the previous autumn (year *t* − 1) because that was when reproduction that gave rise to the spring‐defined cohort occurred (Charlier et al., 2012; Waples, 2005).

### 2.5 Do *N*_b_ estimates from subpopulations within a metapopulation apply to a local (subpopulation) scale and cohort‐specific timescale?

We conducted a series of five tests to address this question. All of these tests address aspects of the spatial scale to which estimates of ${\hat{N}}_{b-\mathrm{LDNe}}$ apply; however, only two of them provide information about the temporal scale of inference (Tests 2 and 3).

#### 2.5.1 Test 1—Between‐subpopulation correlation in ${\hat{N}}_{b-\mathrm{LDNe}}$ time series across cohorts

We predicted that within‐subpopulation time series of ${\hat{N}}_{b-\mathrm{LDNe}}$ should be weakly correlated across subpopulations. Lack of correlation, assuming estimates of ${\hat{N}}_{b-\mathrm{LDNe}}$ reflect true subpopulation *N*_b_, would be consistent with estimates providing a localized (subpopulation‐specific) spatial signal. Correlation between subpopulation time series would not be informative within the context of our analysis because correlation could be due to common environmental effects (see Test 3) on localized estimates or could indicate that estimates apply to a larger scale than subpopulations. We used Spearman's rank correlation tests for each time series of ${\hat{N}}_{b-\mathrm{LDNe}}$ from each of the three subpopulations.

#### 2.5.2 Test 2—Relationship of ${\hat{N}}_{b-\mathrm{LDNe}}$ to family structure

We predicted a positive correlation with cohort‐specific measures of evenness in family size ($\hat{\text{FE}}$) and number of families (${\hat{N}}_{\mathrm{fam}}$). These aspects of family structure should be local in nature and have a large influence on the amount of LD observed. This prediction has a temporal and a spatial component. A positive correlation would be consistent with a localized spatial scale of ${\hat{N}}_{b-\mathrm{LDNe}}$. Lack of correlation could indicate temporal mismatch (effective size estimates apply to longer timescale than the cohort), but not necessarily a spatial scale mismatch (estimates do not apply to subpopulation spatial scale). We used one‐sided Spearman's rank correlation tests because predictions were in one direction.

We examined correlations between ${\hat{N}}_{b-\mathrm{LDNe}}$ and two summary statistics related to family structure. We obtained estimates of the number of full‐sibling families produced (${\hat{N}}_{\mathrm{fam}}$) directly from COLONY. We estimated family evenness ($\hat{\text{FE}}$) as a measure of variance in family size (Whiteley et al., 2013). $\hat{\text{FE}}$ was calculated as $FE=H^{\prime }/H_{\max}^{\prime }$, where $H^{\prime }=-\sum_{1}^{S}p_{i}\text{ln}\left(p_{i}\right)$ and $H_{\max}^{\prime }=\text{ln}\left(S\right)$ (Mulder et al., 2004). *S*, which usually represents the number of species in an evenness calculation, here represented the number of families and *p*_*i*_ represented the proportion comprised of the *i*th family. We chose to use $\hat{\text{FE}}$ because we previously compared $\hat{\text{FE}}$ to variance from a negative binomial distribution fitted to full‐sib family distribution data for summarizing variance in these distributions, and found that $\hat{\text{FE}}$ was more closely related to ${\hat{N}}_{b}$ (Whiteley et al., 2015).

#### 2.5.3 Test 3—Relationship between ${\hat{N}}_{b-\mathrm{LDNe}}$ and an environmental factor (stream flow)

We predicted either an intermediate optimum or positive relationship between ${\hat{N}}_{b-\mathrm{LDNe}}$ and autumn stream flow following Whiteley et al. (2015). We demonstrated a significant quadratic relationship between single‐cohort ${\hat{N}}_{b}$ and autumn stream flow in the portion of this metapopulation with greater connectivity (pooled WB, OS, OL; Whiteley et al., 2015). We hypothesized that increased competition and limitations on available spawning habitat during reproduction at low flows and reduced habitat quality at high flows were responsible for the intermediate optimum relationship (Whiteley et al., 2015). Here, we predict a similar relationship within each subpopulation. This prediction has a temporal and a spatial component. A significant relationship would be consistent with a localized spatial scale of ${\hat{N}}_{b-\mathrm{LDNe}}$. A lack of relationship could indicate a temporal mismatch between ${\hat{N}}_{b-\mathrm{LDNe}}$ and the estimates of mean stream flow, or a spatial scale mismatch.

We used estimates of autumn stream flow from 1 October to 31 December from the autumn prior to the birth of each spring‐defined cohort, following Whiteley et al. (2015). Stream flow was estimated using a flow extension model (Letcher et al., 2016) based on data from a nearby (~10 km) USGS stream gage (Mill River, Northampton, MA, USA) following Xu, Letcher, and Nislow (2010).

#### 2.5.4 Test 4—Robustness of ${\hat{N}}_{b-\mathrm{LDNe}}$ to admixture

We predicted that admixture would not have a large influence on ${\hat{N}}_{b-\mathrm{LDNe}}$ if estimates apply to the subpopulation spatial scale. If admixture did not have a large influence on ${\hat{N}}_{b-\mathrm{LDNe}}$, removing the admixture signal (individuals with high admixture) should have little effect. If admixture did have a large influence on ${\hat{N}}_{b-\mathrm{LDNe}}$, the presence of admixed individuals should elevate LD and depress ${\hat{N}}_{b-\mathrm{LDNe}}$. Therefore, with admixed individuals removed, ${\hat{N}}_{b-\mathrm{LDNe}}$ were expected to increase. We estimated ${\hat{N}}_{b-\mathrm{LDNe}}$ with and without highly admixed individuals (defined as individuals with *q*‐values between .3 and .7 in the STRUCTURE analysis). The difference in ${\hat{N}}_{b-\mathrm{LDNe}}$ with and without highly admixed individuals provided a measure of the strength of the effect of admixture on ${\hat{N}}_{b-\mathrm{LDNe}}$. We chose these cutoff *q*‐values to allow us to filter individuals that were most likely to be admixed and, because of high admixture levels, should have the greatest effect on ${\hat{N}}_{b-\mathrm{LDNe}}$. Individuals with lower *q*‐values have greater uncertainty (Marie, Bernatchez, & Garant, 2011) and in our case would have less of an influence on ${\hat{N}}_{b-\mathrm{LDNe}}$. Our results should be interpreted as a conservative test subject to the limitations of empirical data. An additional caveat is that the removal of incorrectly identified admixed individuals would also be expected to have little effect on ${\hat{N}}_{b-\mathrm{LDNe}}$.

#### 2.5.5 Test 5—Effect of pooling divergent subpopulations on ${\hat{N}}_{b-\mathrm{LDNe}}$

We predicted that ${\hat{N}}_{b-\mathrm{LDNe}}$ that pooled individuals from genetically divergent subpopulations would create mixture LD that would in turn lead to reduced estimates relative to ${\hat{N}}_{b-\mathrm{LDNe}}$ that did not include individuals from divergent subpopulations. Thus, ${\hat{N}}_{b-\mathrm{LDNe}}$ for wider pools of individuals (over an increased spatial scale) might not lead to the predicted increase in ${\hat{N}}_{b-\mathrm{LDNe}}$ that would be expected if the estimates corresponded to metapopulation ${\hat{N}}_{b}$ (Gomez‐Uchida et al., 2013; Palstra & Ruzzante, 2011; Waples & England, 2011). Instead, ${\hat{N}}_{b-\mathrm{LDNe}}$ based on pooled subpopulation samples might be lower than subpopulation‐specific ${\hat{N}}_{b-\mathrm{LDNe}}$. We estimated ${\hat{N}}_{b-\mathrm{LDNe}}$ from four separate pools of individuals corresponding to (i) WB/OS, (ii) WB/OS/OL, (iii) WB/OS/IL, and (iv) WB/OS/OL/IL.

### 2.6 Predicting generational ${\hat{N}}_{e}$ from ${\hat{N}}_{b}$

Waples et al. (2014) provide a novel approach to estimate generational *N*_e_ (the parameter with a richer population genetic foundation) from single‐cohort ${\hat{N}}_{b}$ (the parameter that is more estimable and interpretable) for organisms with overlapping generations. Our second objective was to compare generational ${\hat{N}}_{e}$ derived from cohort‐specific ${\hat{N}}_{b}$ to alternatively obtained empirical estimates of generational ${\hat{N}}_{e}$. We obtained empirical estimates of generational ${\hat{N}}_{e}$ with the Jorde and Ryman approach (Jorde & Ryman, 1995, 1996, 2007) for each subpopulation (hereafter ${\hat{N}}_{e-\mathrm{JR}}$). This is the only unbiased *N*_e_ estimator available for organisms with overlapping generations (Charlier et al., 2012).

To estimate generational ${\hat{N}}_{e}$ based on the approach from Waples et al. (2014), we first calculated adjusted subpopulation‐specific ${\hat{N}}_{b-\mathrm{LDNe}}$ following the equation ${\hat{N}}_{b(\mathrm{Adj})}={\hat{N}}_{b}/\left(1.26-0.323x\left(N_{b}/N_{e}\right)\right)$, where ${\hat{N}}_{b(\mathrm{Adj})}$ were bias‐adjusted values of raw ${\hat{N}}_{b-\mathrm{LDNe}}$, *N*_b_/*N*_e_ was obtained separately for each subpopulation with AgeNe (Waples, Do, & Chopelet, 2011), and ${\hat{N}}_{b}$ were subpopulation‐specific harmonic means of ${\hat{N}}_{b-\mathrm{LDNe}}$. We then divided ${\hat{N}}_{b(\mathrm{Adj})}$ by the ratio of *N*_b_/*N*_e_ from AgeNe to obtain subpopulation‐specific ${\hat{N}}_{e}$ (hereafter ${\hat{N}}_{e(\mathrm{Adj})}$ following the nomenclature of Waples et al. (2014)). For the AgeNe analysis, we used a previously reported life table for WB/OS and OL (Whiteley et al., 2015). We constructed a separate life table for IL based on demographic data reported in Letcher et al. (2014). AgeNe assumes constant population size and stable age structure (Waples et al., 2013) to obtain the *N*b/*N*e ratio. AgeNe implements an index of overdispersion of reproductive success of same‐age, same‐sex individuals termed the Poisson scaling factor (PSF; Waples et al., 2013). We used a PSF of 4.7 for each age class. This value was obtained by first fitting a negative binomial model to full‐sibling family size distributions for each cohort (Whiteley et al., 2015). We used the mean from the fitted negative binomial as an estimate of $\overset{¯}{k}$ and the variance as an estimate of *V*_k_. The ratio $V_{k}/\overset{¯}{k}$ represents an unscaled PSF. We scaled this empirically obtained ratio using equation 3 from Waples (2002),

$$E\left[V_{k2}\right]\approx \;{\overset{¯}{k}}_{2}\;\left[1+{\overset{¯}{k}}_{2}\frac{\left(\frac{V_{k1}}{{\overset{¯}{k}}_{1}}-1\right)}{{\overset{¯}{k}}_{1}}\right]$$

to obtain a PSF $\left(V_{k2}/{\overset{¯}{k}}_{2}\right)$ scaled by the expected $\overset{¯}{k}$ at a constant population size. We used the mean and variance from the fitted negative binomial distributions as ${\overset{¯}{k}}_{1}$ and *V*_k1_, respectively, in this equation. We used the mean of $b_{x}^{\prime }$ values from AgeNe as ${\overset{¯}{k}}_{2}$. The median of the $V_{k2}/{\overset{¯}{k}}_{2}$ ratio across the nine cohorts (4.7) was used as the scaled PSF for AgeNe. We also tested sensitivity of the *N*_b_/*N*_e_ ratio to variation in the PSF using values of 1, 2, 4, and 8 for this parameter.

We compared ${\hat{N}}_{e(\mathrm{Adj})}$ obtained with the Waples et al. (2014) approach to empirical estimates of ${\hat{N}}_{e}$ based on the Jorde and Ryman approach (Jorde & Ryman, 1995, 1996, 2007). We used the program GONe (Coombs, Letcher, & Nislow, 2012) to implement the Jorde and Ryman approach to estimate generational ${\hat{N}}_{e-\mathrm{JR}}$. This approach assumes there is a generational *N*_e_ for each subpopulation that is stable over time. We obtained an estimate of generational ${\hat{N}}_{e-\mathrm{JR}}$ for the entire period based on the harmonic mean of the 36 values of ${\hat{N}}_{e-\mathrm{JR}}$, one for each pair of cohorts (consecutive and nonconsecutive) in the interval 2001–2009. We used the same life tables and the program GONe to calculate the correction factor for overlapping generations (*C*) and generation interval (*G*) to correct for age structure effects on allele frequency shifts (Jorde, 2012; Jorde & Ryman, 1995). The parameters *C* and *G* appear to be robust to uncertainty in life table parameters (Jorde & Ryman, 1995). We used sample plan I because fish were released after capture, which requires the estimated sample size of newborns (*N*_1_) entering the population for the first year under consideration (Jorde, 2012). We chose to use the mean of estimates of YOY from 2002 to 2009 (years for which estimates were available; WB/OS = 519, OL = 178, IL = 195) because we used every possible combination of cohorts to estimate ${\hat{N}}_{e-\mathrm{JR}}$.

We also addressed the question: How many consecutive cohorts need to be sampled to apply the Waples et al. (2014) approach for these populations of brook trout? First, we tested the number of consecutive cohorts needed to reliably estimate harmonic ${\hat{N}}_{b}$ of populations. We calculated the harmonic mean for all possible overlapping successive subsets for consecutive cohorts of size *N *=* *2 through *N *=* *9 cohorts (the single value obtained for *N *=* *9 cohorts was the same as the overall harmonic mean). For example, there were eight possible overlapping consecutive cohorts of size two and seven possible overlapping consecutive cohorts of size three. Once these subsets of harmonic mean ${\hat{N}}_{b-\mathrm{LDNe}}$ were obtained, we applied the Waples et al. (2014) approach to obtain ${\hat{N}}_{e(\mathrm{Adj})}$ for each subset of consecutive cohorts.

## 3 Results

### 3.1 Population structure

The STRUCTURE analysis of YOY subsampled by full‐sib family membership provided strong evidence for three subpopulations (WB & OS, OL, and IL; Fig. S1). The Evanno approach provided the most support for *K *=* *2 in four replicates and *K *=* *3 in six replicates. Mean LnP(*D*) values increased substantially from *K *=* *2 to *K *=* *3 (mean increase in LnP(*D*) across 10 replicates was 928.8) but increased slightly from *K *=* *3 to *K *=* *4 (mean increase in LnP(*D*) across 10 replicates was 205.7; Fig. S1). Assignments associated with a fourth cluster for the *K *=* *4 models were not associated with a specific geographic location or cohort and were not biologically plausible (data not shown). We therefore used *K *=* *3 to define subpopulations for subsequent analyses.

**Figure 2:**
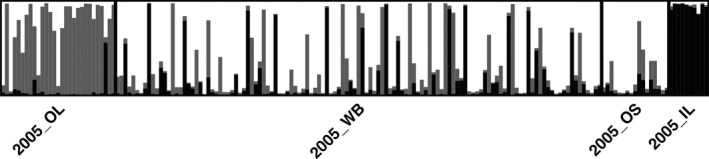
Proportion of the genome (*q*) of each individual assigned by STRUCTURE to each subpopulation within a Massachusetts metapopulation. Results are shown for the *K *=* *3 model for a representative (2005) cohort; all cohorts are shown in Fig. S2. This analysis is based on one YOY randomly selected per full‐sib family. Each bar (column) represents one individual sampled in 2005 from OL, WB, OS, or IL

Patterns among the 10 replicates for *K *=* *3 were highly consistent, demonstrating that the full‐sib subsampling procedure did not influence inference of *K*. In all replicates, *K *=* *3 revealed a clearly differentiated IL subpopulation, a differentiated OL subpopulation, and a combined WB and OS (hereafter WB/OS) subpopulation (Figure 2; Fig. S2). There was substantial evidence for movement (defined as individuals with *q*‐values >.70 and assigned to a location different from the sample location) between OL and WB/OS (Table 1). There was also substantial evidence of movement out of but not into IL (Table 1; Figure 2, Fig. S2). Genetically admixed individuals (defined as individuals with *q*‐values between .3 and .7) occurred in all locations, but with dramatically lower frequency in IL (Figure 2, Fig. S2). Mean estimated admixture across cohorts for WB/OS was 0.19, OL was 0.24, and IL was 0.06.

**Table 1:** Estimated movement rates of individual brook trout among subpopulations of the West Brook. Estimates are based on STRUCTURE analysis of either only age‐0 (YOY) individuals (YOY‐only) or for all individuals regardless of age at first capture (all individuals). For the YOY‐only analysis, one full‐sib per family was randomly chosen to avoid biased inference with STRUCTURE. Zero values with an underline are true zeros, and others are small values above 0 (<0.001). Values are means of estimates across cohorts

	From WB/OS	
	To OL	To IL
YOY‐only	0.30	0
All individuals	0.34	0

	From OL	
	To WB/OS	To IL
YOY‐only	0.10	0
All individuals	0.11	0

	From IL	
	To WB/OS	To OL
YOY‐only	0.06	0.01
All individuals	0.05	0.02

We randomly chose one of the 10 STRUCTURE replicates to test for conformation to HW proportions and LD with the YOY‐only and sibship subsampled data set after assigning individuals to one of the three subpopulations. 24 of 310 (7.7%) tests for deviations from HW proportions within cohorts and subpopulations were significant (*p *<* *.05), where 16 were expected by chance. Nine tests remained significant following sequential Bonferroni correction for 12 tests within each subpopulation–cohort combination. There were no patterns across loci or subpopulations for tests that remained significant following correction for multiple tests. One hundred and two of 1662 (6.1%) tests for LD were significant (*p *<* *.05), where 83 were expected by chance. Six tests remained significant following sequential Bonferroni correction for 66 tests within each subpopulation–cohort combination. There were no patterns across loci or subpopulations for tests that remained significant.

We performed a second STRUCTURE analysis on the entire data set to allow us to assign individuals of all ages to natal subpopulations. This STRUCTURE analysis with all individuals included and with *K *=* *3 yielded quantitatively and qualitatively similar patterns to the analysis based on one randomly subsampled full‐sib YOY per family. For the individuals randomly chosen in the YOY‐only analysis, the mean difference in *q*‐values for the first cluster was .003, the second cluster was .008, and the third cluster was .005, the overall mean difference in *q*‐values for both STRUCTURE analyses across clusters of .005.

We used the complete data set, following STRUCTURE‐based assignment of individuals with *q*‐values >.70 to putative natal locations, for all subsequent analyses. Within‐subpopulations allelic diversity and heterozygosity were similar among cohorts (Table 2). Genetic diversity was greatest in WB/OS (mean AR = 7.1, *H*_S_ = 0.628) and OL (mean AR = 5.6, *H*_S_ = 0.540), and lowest in IL (mean AR = 3.8, *H*_S_ = 0.438; Table 2). The mean number of alleles per locus, averaged across nine cohorts, ranged from 3.2 to 17.9 in WB/OS, 3.3 to 17.3 in OL, and 3.4 to 17.1 in IL (Table S1).

**Table 2:** Genetic summary statistics for 12 microsatellite loci for brook trout cohorts from the West Brook subpopulations. Subpopulations (WB/OS, OL, and IL) were defined through STRUCTURE analysis. Cohorts are defined by the year of emergence. *S* is the number of individuals genotyped per cohort. *AR* is mean allelic richness, based on the minimum sample size of 53 individuals. *H*_S_ is mean expected heterozygosity. *F*_IS_ is a measure of departure from HW proportions. ${\hat{N}}_{\mathrm{fam}}$ is the number of estimated full‐sib families. $\hat{\text{FE}}$ is family evenness, a measure inversely related to variance in the full‐sib family distribution of each cohort. ${\hat{N}}_{b-\mathrm{LDNe}}$ (shown with 95% CI) is the effective number of breeders estimated for combined individuals from WB/OS, OL, and IL with the program LDNe (assuming random mating). ${\hat{N}}_{C}$ (shown with 95% CI) is the number of adults (age‐1 and older) estimated from the fall previous to the listed spring‐defined cohort. ${\hat{N}}_{b-\mathrm{LDNe}}/{\hat{N}}_{C}$ is the ratio of both measures

Cohort	*S*	AR	*H*_S_	*F*_IS_	${\hat{N}}_{\mathrm{fam}}$	$\hat{\text{FE}}$	${\hat{N}}_{b-\mathrm{LDNe}}$	${\hat{N}}_{C}$	${\hat{N}}_{b-\mathrm{LDNe}}/{\hat{N}}_{C}$
West Brook & Open‐Small (WB/OS)									
2001	636	7.0	0.632	0.023	202	0.910	68.8 (60.2–78.5)	–	–
2002	484	7.4	0.635	0.061	114	0.856	25.1 (21.4–29.2)	–	–
2003	734	7.2	0.640	0.007	225	0.915	75.5 (61.0–92.8)	846.7 (507.0, 2247.1)	0.09
2004	587	7.0	0.627	0.008	198	0.898	51.6 (43.5–60.8)	493.6 (446.7, 564.8)	0.11
2005	431	7.3	0.633	0.015	180	0.926	83.6 (69.1–101.3)	338.3 (312.9, 370.0)	0.25
2006	352	6.7	0.608	−0.011	89	0.834	22.6 (17.9–28.0)	388.2 (354.9, 441.9)	0.06
2007	186	7.2	0.630	0.008	89	0.945	61.6 (45.8–84.6)	256.5 (225, 298.4)	0.24
2008	379	7.4	0.631	0.001	119	0.918	50.2 (41.4–60.6)	129.0 (114.6, 156.6)	0.39
2009	567	6.9	0.619	0.010	133	0.916	48.5 (39.4–59.1)	116.7 (107.2, 136.6)	0.42
Open‐Large (OL)									
2001	251	5.2	0.521	−0.004	101	0.945	90.4 (61.7–138.3)	–	–
2002	113	5.4	0.542	−0.025	44	0.906	35.2 (24.0–53.0)	–	–
2003	149	5.7	0.554	−0.042	48	0.838	25.2 (16.8–37.3)	228.6 (111.1, 971.1)	0.11
2004	199	5.5	0.526	0.010	81	0.950	64.0 (40.8–105.3)	210.7 (178.4, 253.8)	0.30
2005	129	5.7	0.532	−0.004	63	0.954	72.5 (44.6–134.0)	132.2 (119.1, 152.2)	0.55
2006	60	5.8	0.518	0.019	27	0.949	98.5 (37.5‐∞)	191.4 (171.9, 224.7)	0.52
2007	91	5.8	0.543	−0.007	53	0.953	53.9 (28.7–128.4)	96.3 (82.4, 114.9)	0.56
2008	121	5.1	0.591	−0.035	32	0.678	7.1 (3.8–10.6)	91.0 (79.0, 108.7)	0.08
2009	147	5.8	0.535	0.000	58	0.946	62.3 (45.0–89.6)	91.3 (75.1, 120.5)	0.68
Isolated‐Large (IL)									
2001	239	3.7	0.427	−0.032	51	0.935	38.9 (28.0–53.7)	–	–
2002	224	3.5	0.436	0.021	41	0.902	23.1 (14.6–35.0)	–	–
2003	278	3.6	0.418	−0.036	51	0.905	34.8 (22.1–53.3)	179.3 (93.0, 605.6)	0.19
2004	148	3.8	0.445	0.033	38	0.932	50.2 (33.2–79.1)	164.4 (146.4, 187.4)	0.31
2005	77	3.9	0.426	−0.005	27	0.930	77.0 (35.9–353.4)	173.4 (153.8, 198.9)	0.44
2006	53	4.1	0.448	−0.089	19	0.948	31.3 (17.6–66.0)	110.2 (100.7, 130.1)	0.28
2007	76	4.2	0.472	−0.037	25	0.938	58.6 (29.8–170.3)	62.8 (57.1, 72.0)	0.93
2008	67	3.6	0.423	0.021	20	0.942	37.1 (18.5–96.9)	53.1 (46.5, 65.7)	0.70
2009	154	3.9	0.449	−0.032	35	0.915	39.4 (24.3–65.6)	51.2 (43.9, 63)	0.77

All of the 27 pairwise tests for genic differentiation were significant based on Fisher's method and following sequential Bonferroni correction either for the three tests within each cohort (nominal *p *=* *.017) or for all 27 tests across the nine cohorts (nominal *p *=* *.0019). Overall *F*_ST_ was 0.085 (95% CI 0.059–0.116) and overall $F_{\mathrm{ST}}^{\prime }$ was 0.182 (95% CI 0.116–0.260). WB/OS and OL were the least genetically differentiated (mean pairwise *F*_ST_ = 0.06, $F_{\mathrm{ST}}^{\prime }$ = 0.16), followed by WB/OS and IL (mean pairwise *F*_ST_ = 0.09, $F_{\mathrm{ST}}^{\prime }$ = 0.16), and OL and IL (mean pairwise *F*_ST_ = 0.18, $F_{\mathrm{ST}}^{\prime }$ = 0.35; Table 3; Table S2). PCA revealed clearly differentiated clusters of cohorts within each subpopulation (Fig. S3). Allele frequency divergence among cohorts within subpopulations was greatest in IL ($F_{S}^{\prime }$ = 0.037), followed by OL ($F_{S}^{\prime }$ = 0.035), and WB/OS ($F_{S}^{\prime }$ = 0.021).

**Table 3:** Mean (2001–2009 cohorts) pairwise *F*_ST_ (Nei's *G*_ST_, above diagonal) and pairwise $F_{\mathrm{ST}}^{\prime }$ ($G_{\mathrm{ST}}^{\prime \prime }$, below diagonal) for three subpopulations (OL, WB/OS, and IL) with the West Brook study system. Estimates are based on entire cohorts following STRUCTURE‐based assignments to putative natal locations for each individual. Mean sample size across cohorts is shown in parentheses in column headings

	OL (140)	WB/OS (484)	IL (146)
OL	–	0.06	0.18
WB/OS	0.16	–	0.09
IL	0.35	0.20	–

### 3.2 ${\hat{N}}_{b}$ and ${\hat{N}}_{C}$ over time within subpopulations

**Figure 3:**
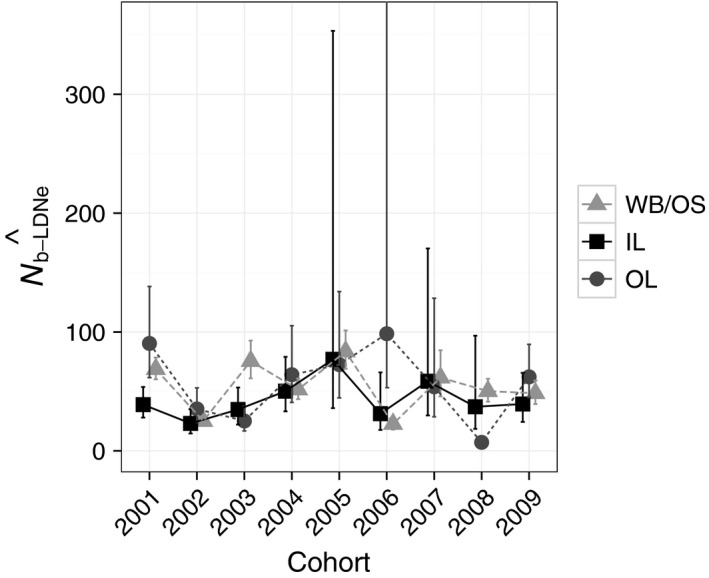
${\hat{N}}_{b-\mathrm{LDNe}}$ within subpopulations of a Massachusetts brook trout metapopulation based on entire cohort samples. ${\hat{N}}_{b-\mathrm{LDNe}}$ is shown separately for WB/OS (gray triangles), OL (gray circles), and IL (black squares) for each cohort (*x*‐axis). 95% confidence intervals are based on jackknifing over individuals. The upper limit on the confidence interval for OL in 2006 was infinity, and all other upper confidence limits were finite

We estimated ${\hat{N}}_{b-\mathrm{LDNe}}$ based on entire reconstructed cohorts and following STRUCTURE reassignments for each subpopulation. The harmonic mean of point estimates for ${\hat{N}}_{b-\mathrm{LDNe}}$ across the nine cohorts for WB/OS (${\hat{N}}_{b-\mathrm{LDNe}-\mathrm{WB}/\mathrm{OS}}$) was 45.0, ${\hat{N}}_{b-\mathrm{LDNe}-\mathrm{OL}}$ was 30.6, and ${\hat{N}}_{b-\mathrm{LDNe}-\mathrm{IL}}$ was 38.8 (Table 2; Figure 3). There was substantial overlap of 95% confidence intervals across subpopulations in five of the nine cohorts (Figure 3). Only one confidence interval had an upper limit of infinity (OL, 2006; Figure 3). ${\hat{N}}_{b-\mathrm{LDNe}-\mathrm{OL}}$ point estimates were more variable (CV = 0.98) than either ${\hat{N}}_{b-\mathrm{LDNe}-\mathrm{WB}/\mathrm{OS}}$ (CV = 0.46) or ${\hat{N}}_{b-\mathrm{LDNe}-\mathrm{IL}}$ (CV = 0.46).

**Figure 4:**
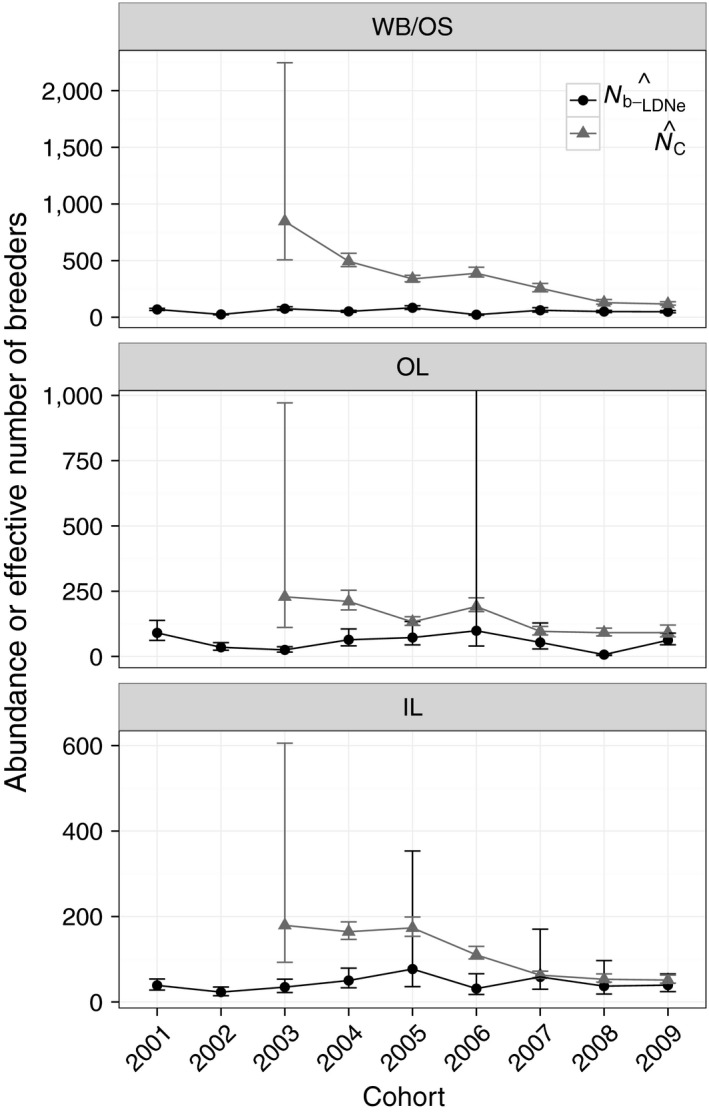
Estimates of ${\hat{N}}_{b-\mathrm{LDNe}}$ and adult abundance (${\hat{N}}_{C}$) over time within the three subpopulations of a Massachusetts brook trout metapopulation. ${\hat{N}}_{b-\mathrm{LDNe}}$ is shown with black‐filled circles. ${\hat{N}}_{C}$ is shown with gray triangles. ${\hat{N}}_{C}$ is lagged by 1 year relative to the spring‐defined birth year of cohorts; that is, ${\hat{N}}_{C}$ represents the fall from the year prior to the year shown on the *x*‐axis. 95% confidence intervals are based on jackknifing over individuals. The upper limit on the confidence interval for OL in 2006 was infinity, and all other upper confidence limits were finite

${\hat{N}}_{C}$ followed a declining trend in each subpopulation (Table 2; Figure 4). ${\hat{N}}_{b-\mathrm{LDNe}}$ were stable relative to ${\hat{N}}_{C}$ for each subpopulation (Figure 4). ${\hat{N}}_{b-\mathrm{LDNe}}/{\hat{N}}_{C}$ based on the ratio of harmonic means of both ${\hat{N}}_{b-\mathrm{LDNe}}$ and ${\hat{N}}_{C}$ across cohorts was 0.19 (WB/OS), 0.24 (OL), and 0.45 (IL). Cohort‐specific ${\hat{N}}_{b-\mathrm{LDNe}}/{\hat{N}}_{C}$ ratios ranged from 0.06 to 0.42 in WB/OS (mean = 0.22), 0.08 to 0.68 in OL (mean = 0.40), and 0.19 to 0.77 in IL (mean = 0.51; Table 2).

### 3.3 Do *N*_e_ and *N*_b_ estimates from subpopulations within a metapopulation apply to a local (subpopulation) scale and cohort‐specific timescale?

#### 3.3.1 Test 1

There was little temporal synchrony among ${\hat{N}}_{b-\mathrm{LDNe}}$ for WB/OS, OL, or IL (WB/OS–OL, ρ = −.02, *p *=* *.98; WB/OS–IL, ρ = .67, *p *=* *.08; OL–IL, ρ = .19, *p *=* *.66; Figure 3).

#### 3.3.2 Test 2

The two measures we used to summarize family structure (${\hat{N}}_{\mathrm{fam}}$ and $\hat{\text{FE}}$) were significantly correlated with variation in ${\hat{N}}_{b-\mathrm{LDNe}}$ in WB/OS. ${\hat{N}}_{b-\mathrm{LDNe}-\mathrm{WB}/\mathrm{OS}}$ were positively correlated with $\hat{\text{FE}}$ (ρ = .67, *p *=* *.04) and ${\hat{N}}_{\mathrm{fam}}$ (ρ = .65, *p *=* *.04). For OL, ${\hat{N}}_{b-\mathrm{LDNe}-\mathrm{OL}}$ were positively correlated with $\hat{\text{FE}}$ (ρ = .62, *p *=* *.06) and ${\hat{N}}_{\mathrm{fam}}$ (ρ = .29, *p *=* *.25), although neither was significant. For both WB/OS and OL, the lowest values of ${\hat{N}}_{b-\mathrm{LDNe}}$ (<approx. 50) were observed when both $\hat{\text{FE}}$ *and* ${\hat{N}}_{\mathrm{fam}}$ were low (Table 2, Fig. S4). ${\hat{N}}_{b-\mathrm{LDNe}-\mathrm{WB}/\mathrm{OS}}$ reached the lowest values in the 2002 and 2006 cohorts, when low values of $\hat{\text{FE}}$ combined with low values of ${\hat{N}}_{\mathrm{fam}}$ (Table 2; Fig. S4). This combined effect of low $\hat{\text{FE}}$ and ${\hat{N}}_{\mathrm{fam}}$ was also observed in OL. The 2008 cohort had very low $\hat{\text{FE}}$ (0.678), and among the lowest ${\hat{N}}_{\mathrm{fam}}$ (32), and had the lowest ${\hat{N}}_{b-\mathrm{LDNe}-\mathrm{OL}}$ (7.1). The 2003 cohort had the second lowest ${\hat{N}}_{b-\mathrm{LDNe}-\mathrm{OL}}$ (25.2), the second lowest $\hat{\text{FE}}$ (0.838), and below average ${\hat{N}}_{\mathrm{fam}}$ (48; mean = 56.3).

The relationship between ${\hat{N}}_{b-\mathrm{LDNe}-\mathrm{IL}}$ and family structure was weaker. $\hat{\text{FE}}$ ranged from 0.902 to 0.948 (Table 2), providing evidence for lower reproductive variance in IL compared to WB/OS or OL. ${\hat{N}}_{b-\mathrm{LDNe}-\mathrm{IL}}$ ranged from 19 to 51 (Table 2). ${\hat{N}}_{b-\mathrm{LDNe}-\mathrm{IL}}$ were positively, but not significantly, correlated with $\hat{\text{FE}}$ (ρ = .10, *p *=* *.42), and negatively, but not significantly, correlated with ${\hat{N}}_{\mathrm{fam}}$ (ρ = −.07, *p *=* *.57). Unlike WB/OS and OL, the lowest values of ${\hat{N}}_{b-\mathrm{LDNe}-\mathrm{IL}}$ were associated with either low $\hat{\text{FE}}$ or ${\hat{N}}_{\mathrm{fam}}$ (Table 2; Figure 3, Fig. S4). For example, the 2002 cohort had the lowest ${\hat{N}}_{b-\mathrm{LDNe}-\mathrm{IL}}$ (23.1) and the lowest $\hat{\text{FE}}$ (0.902) but higher than average ${\hat{N}}_{\mathrm{fam}}$ (41, mean = 34.1). The 2006 cohort had the second lowest ${\hat{N}}_{b-\mathrm{LDNe}-\mathrm{IL}}$ (31.3), the highest $\hat{\text{FE}}$ (0.948), but the lowest ${\hat{N}}_{\mathrm{fam}}$ (19).

#### 3.3.3 Test 3

**Figure 5:**
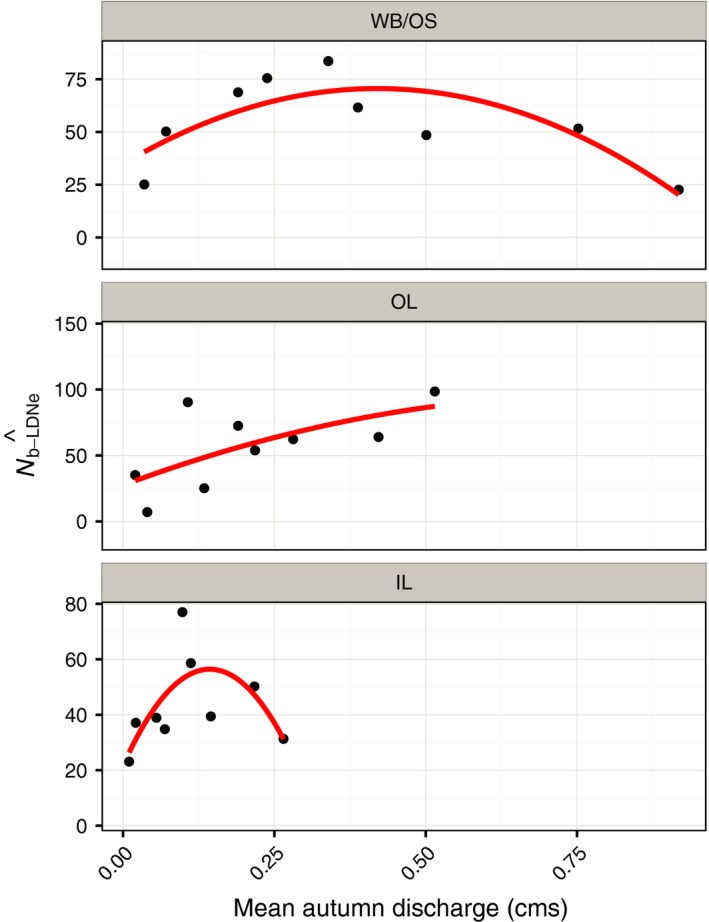
Relationship between autumn stream flow (discharge) and ${\hat{N}}_{b-\mathrm{LDNe}}$ in subpopulations within a Massachusetts brook trout metapopulation. Mean autumn discharge is the average of mean daily discharge taken from 1 October to 31 December (when reproduction occurred) in the year preceding a spring‐born cohort. The regression lines show a fitted linear model with quadratic term

**Figure 6:**
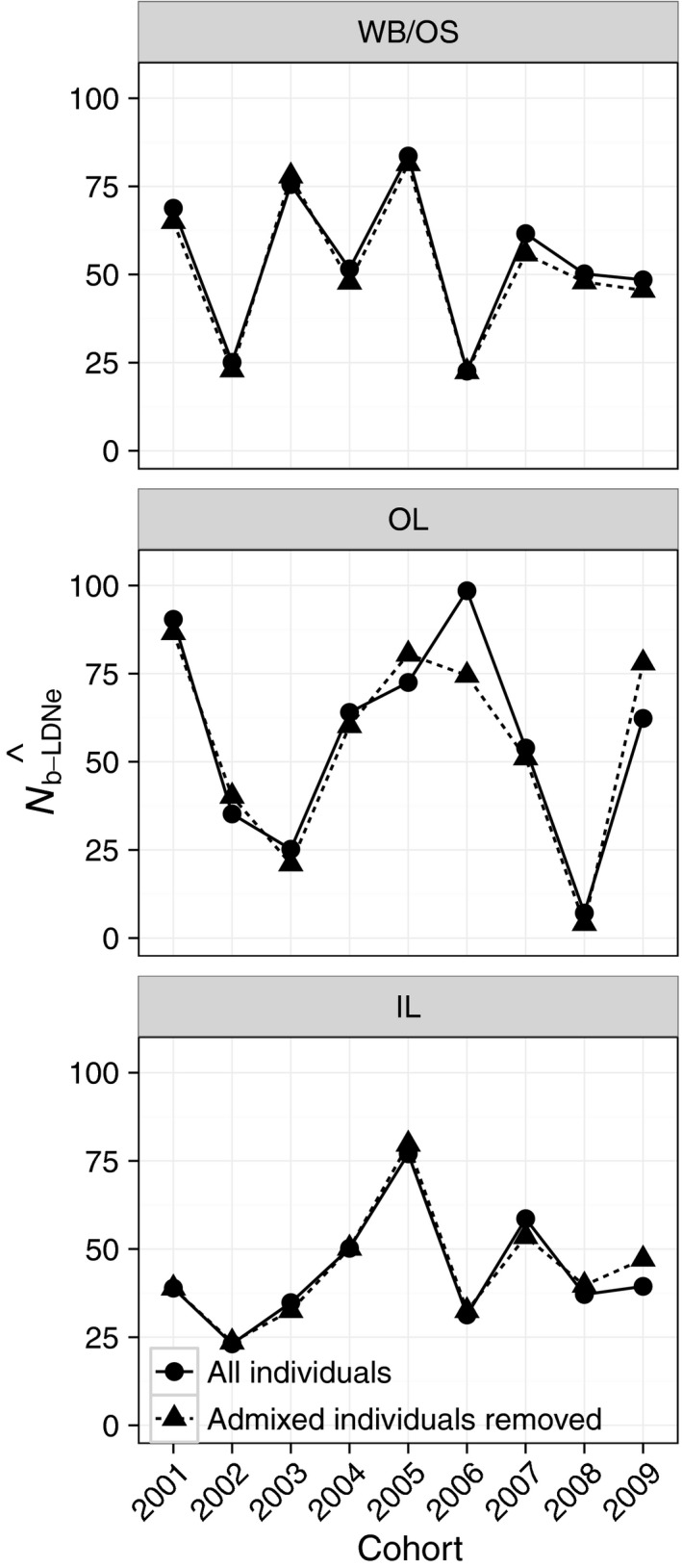
The effect of admixture on ${\hat{N}}_{b-\mathrm{LDNe}}$. Shown are estimates of ${\hat{N}}_{b-\mathrm{LDNe}}$ with all individuals (solid lines connecting circles) from each cohort or with admixed (.3 < *q *<* *.7) individuals removed (dashed lines connecting triangles) for each of the three subpopulations

There was a significant (*p *=* *.04) quadratic relationship between point estimates of ${\hat{N}}_{b-\mathrm{LDNe}-\mathrm{WB}/\mathrm{OS}}$ and autumn stream flow (Table 4; Figure 5). The quadratic term in the model was significant (*p *=* *.02) and the quadratic model explained 66% of the variation (Table 4). ${\hat{N}}_{b-\mathrm{LDNe}-\mathrm{OL}}$ increased linearly with autumn stream flow for OL (Figure 6; *p *=* *.06). There was limited and nonsignificant evidence for a quadratic relationship between ${\hat{N}}_{b-\mathrm{LDNe}-\mathrm{IL}}$ and autumn stream flow (*p *=* *.14, *R* ^2^ = .48, quadratic term *p *=* *.06; Table 4; Figure 5).

**Table 4:** Tests for stream flow effects on ${\hat{N}}_{b-\mathrm{LDNe}}$ in three brook trout subpopulations. ${\hat{N}}_{b-\mathrm{LDNe}}$ was the response variable. Autumn flow (1 October to 31 December) with or without a quadratic term in the linear model was used as a predictor. Quadratic terms were added to test for intermediate optima. Shown are *F*‐values (*F*), *p*‐values (*p*), degrees of freedom (*df*), and proportion of variance explained (*R* ^2^) separately for the three subpopulations (WB/OS, OL, and IL)

Predictors	${\hat{N}}_{b-\mathrm{LDNe}}$ (response variable)			
	*F*	*p*	*df*	*R* ^2^
WB/OS				
Autumn	0.63	0.45	1,7	.08
Autumn quadratic	5.70	0.04	2,6	.66
OL				
Autumn	5.00	0.06	1,7	.41
Autumn quadratic	2.20	0.19	2,6	.42
IL				
Autumn	0.13	0.72	1,7	.02
Autumn quadratic	2.73	0.14	2,6	.48

#### 3.3.4 Test 4

Mean estimated admixture across cohorts was 0.13 (WB/OS), 0.16 (OL), and 0.03 (IL; Table S4). We removed admixed individuals to test for an effect on ${\hat{N}}_{b-\mathrm{LDNe}}$. Only four of 27 cohorts across the three subpopulations provided weak evidence that the inclusion of admixed individuals depressed ${\hat{N}}_{b-\mathrm{LDNe}}$ (Figure 6). For WB/OS, there was a decrease (opposite of predicted effect) in ${\hat{N}}_{b-\mathrm{LDNe}-\mathrm{WB}/\mathrm{OS}}$ in eight of nine cohorts when admixed individuals were excluded. The mean change to ${\hat{N}}_{b-\mathrm{LDNe}}$ was −2.9 (Table S4, Figure 6). This reduction likely occurred through reduction in ${\hat{N}}_{\mathrm{fam}}$ when admixed individuals were removed (mean reduction was 16.2; Table S4). Removing admixed individuals had a minor effect on $\hat{\text{FE}}$ and the direction of this effect varied (five of nine led to smaller $\hat{\text{FE}}$; mean absolute value of change = 0.003). Effects of removing admixed individuals in OL were more mixed (Figure 6). Removal of admixed individuals led to a reduction in ${\hat{N}}_{b-\mathrm{LDNe}-\mathrm{OL}}$ for 67% of the cohorts (6 of 9; Figure 6). The three cohorts with a positive response in ${\hat{N}}_{b-\mathrm{LDNe}}$ were consistent with LD induced by the presence of admixed individuals. There was a reduction in ${\hat{N}}_{\mathrm{fam}}$ in all three of these cohorts, and in two of these cases, $\hat{\text{FE}}$ also decreased. In the absence of an effect on admixture on LD, removal of admixed individuals should have led to a decrease in ${\hat{N}}_{b-\mathrm{LDNe}-\mathrm{OL}}$ in these cases. In IL, the proportion of admixture was small and removal of admixed individuals tended to have a small effect on ${\hat{N}}_{b-\mathrm{LDNe}-\mathrm{IL}}$ (mean value of change to ${\hat{N}}_{b-\mathrm{LDNe}-\mathrm{IL}}$ was 0.8; Figure 6). The change was <3 in seven of nine cohorts. Only the 2009 cohort was consistent with the predicted effect of LD caused by admixture. In this cohort, ${\hat{N}}_{b-\mathrm{LDNe}-\mathrm{IL}}$ were greater by a mean of 7.7 upon removal of admixed individuals, despite a decrease in $\hat{\text{FE}}$ and no effect on ${\hat{N}}_{\mathrm{fam}}$ (Table S4).

#### 3.3.5 Test 5

**Figure 7:**
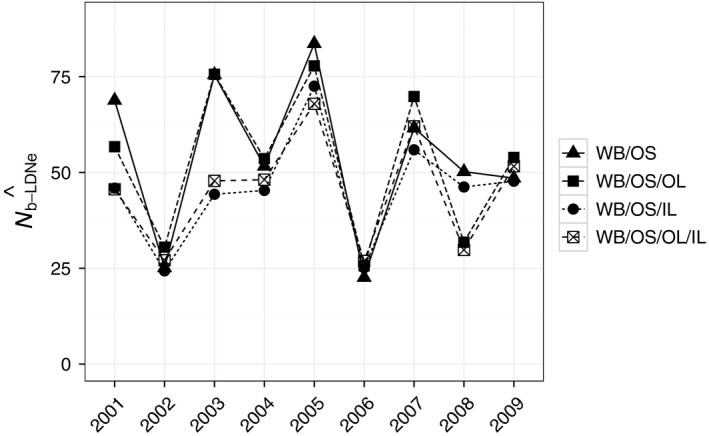
The effect of population subdivision on ${\hat{N}}_{b-\mathrm{LDNe}}$. All individuals from WB/OS cohorts were used to estimate ${\hat{N}}_{b-\mathrm{LDNe}}$ (WB/OS). Individuals from divergent subpopulations OL and IL were added separately or jointly to WB/OS to test for an effect of mixing populations on ${\hat{N}}_{b-\mathrm{LDNe}}$ for each cohort

We observed a reduction in ${\hat{N}}_{b-\mathrm{LDNe}}$, likely due to mixture LD, when individuals from IL were pooled with individuals from WB/OS, regardless of whether individuals from OL were also included (Figure 7). The harmonic mean across cohorts did not differ for ${\hat{N}}_{b-\mathrm{LDNe}-\mathrm{WB}/\mathrm{OS}}$ and ${\hat{N}}_{b-\mathrm{LDNe}-\mathrm{WB}/\mathrm{OS}/\mathrm{OL}}$, and was smaller for both ${\hat{N}}_{b-\mathrm{LDNe}-\mathrm{WB}/\mathrm{OS}/\mathrm{IL}}$ and ${\hat{N}}_{b-\mathrm{LDNe}-\mathrm{WB}/\mathrm{OS}/\mathrm{OL}/\mathrm{IL}}$ (Table 5). The mean difference across cohorts between ${\hat{N}}_{b-\mathrm{LDNe}-\mathrm{WB}/\mathrm{OS}/\mathrm{OL}}$ and ${\hat{N}}_{b-\mathrm{WB}/\mathrm{OS}}$ was −1.4, despite a mean increase in $\hat{\text{FE}}$ of 0.006 and a mean addition of 40.0 to ${\hat{N}}_{\mathrm{fam}}$ (Table S5, Fig. S5). This is consistent with minor mixture LD that canceled out the positive effects (on ${\hat{N}}_{b-\mathrm{LDNe}}$) of adding families and increasing family evenness. The mean difference across cohorts between ${\hat{N}}_{b-\mathrm{LDNe}-\mathrm{WB}/\mathrm{OS}/\mathrm{IL}}$ and ${\hat{N}}_{b-\mathrm{LDNe}-\mathrm{WB}/\mathrm{OS}}$ was −8.9, despite a mean increase in $\hat{\text{FE}}$ of 0.013 and a mean addition of 29.8 to ${\hat{N}}_{\mathrm{fam}}$ (Table S5, Fig. S5). Similarly, the mean difference across cohorts between ${\hat{N}}_{b-\mathrm{LDNe}-\mathrm{WB}/\mathrm{OS}/\mathrm{OL}/\mathrm{IL}}$ and ${\hat{N}}_{b-\mathrm{WB}/\mathrm{OS}}$ was −9.0, despite a mean increase in $\hat{\text{FE}}$ of 0.016 and a mean addition of 68.6 to ${\hat{N}}_{\mathrm{fam}}$ (Table S5, Fig. S5). This is consistent with strong mixture LD when more divergent genotypes from IL were pooled with individuals from the connected part of the system and this strong mixture LD overwhelmed the positive effects (on ${\hat{N}}_{b-\mathrm{LDNe}}$) of adding families and increasing family evenness.

**Table 5:** Effect of addition of genetically divergent populations on subpopulation‐specific estimates of ${\hat{N}}_{b-\mathrm{LDNe}}$. Harmonic means of estimates are shown for the three subpopulations (WB/OS, OL, and IL) and for the addition of genetically divergent populations to WB/OS (WB/OS/OL, WB/OS/IL, and WB/OS/OL/IL), where subpopulations added to WB/OS are separated by a “/.” 95% confidence intervals are shown in parentheses. Shown are the harmonic mean of the point estimates, and lower and upper confidence intervals across the nine cohorts

Subpopulation	${\hat{N}}_{b-\mathrm{LDNe}}$
WB/OS	45.0 (38.9–51.9)
OL	30.6 (22.6–40.1)
IL	38.8 (24.5–64.3)
WB/OS/OL	45.3 (39.3–51.9)
WB/OS/IL	40.6 (35.1–46.7)
WB/OS/OL/IL	40.6 (35.6–46.1)

### 3.4 Generational ${\hat{N}}_{e}$

Mean ${\hat{N}}_{e-\mathrm{JR}}$ across all nine cohorts for WB/OS was 62.9 (95% CI: 47.7–80.2), for OL was 39.1 (95% CI: 27.7–52.5), and for IL was 41.6 (95% CI: 25.9–61.0; Table 6). The ratio of the subpopulation‐specific harmonic mean of ${\hat{N}}_{b-\mathrm{LDNe}}$ to the overall ${\hat{N}}_{e-\mathrm{JR}}$ was 0.72 for WB/OS, 0.78 for OL, and 0.93 for IL (Table 6). The *N*_b_/*N*_e_ ratio from AgeNe varied with the choice of PSF. This ratio ranged from 0.59 to 0.70 for WB/OS and OL and 0.63–0.70 for IL when the PSF ranged from 1 to 8. We used a PSF of 4.7 based on empirical estimates of variance in family size in this study system for subsequent calculations. This PSF corresponded to an *N*_b_/*N*_e_ ratio of 0.63 for WB/OS and OL and 0.58 for IL. ${\hat{N}}_{e(\mathrm{Adj})}$ from the Waples et al. (2014) approach calculated based on the harmonic mean of ${\hat{N}}_{b-\mathrm{LDNe}}$ for each subpopulation (across the nine cohorts) was greater than harmonic mean ${\hat{N}}_{e-\mathrm{JR}}$ by 7% (WB/OS), 18% (OL), and 50% (IL; Table 6).

**Table 6:** Empirical estimation of generational ${\hat{N}}_{e}$ for three brook subpopulations. ${\hat{N}}_{b-\mathrm{LDNe}}$ were estimated for each cohort in each subpopulation with the program LDNe. ${\hat{N}}_{e(\mathrm{Adj})}$ was calculated with the Waples et al. (2014) approach based on subpopulation‐specific harmonic means of ${\hat{N}}_{b-\mathrm{LDNe}}$ (see Methods). A Poisson scaling factor of 4.7 was used to calculate the *N*_b_/*N*_e_ ratio with AgeNe. ${\hat{N}}_{e-\mathrm{JR}}$ are estimates of generational *N*_e_ obtained with the Jorde and Ryman approach (Jorde & Ryman, 1995). The ratios of the harmonic mean of ${\hat{N}}_{b-\mathrm{LDNe}}$ to ${\hat{N}}_{e-\mathrm{JR}}$ and predicted generational ${\hat{N}}_{e}$ to ${\hat{N}}_{e-\mathrm{JR}}$ are also shown

Cohort	${\hat{N}}_{b-\mathrm{LDNe}}$	${\hat{N}}_{e(\mathrm{Adj})}$	${\hat{N}}_{e-\mathrm{JR}}$	${\hat{N}}_{b-\mathrm{LDNe}}/{\hat{N}}_{e-\mathrm{JR}}$	${\hat{N}}_{e(\mathrm{Adj})}/{\hat{N}}_{e-JR}$
WB/OS	45.0	67.6	62.9	0.72	1.08
OL	30.6	46.0	39.1	0.78	1.18
IL	38.8	62.4	41.6	0.93	1.50

**Figure 8:**
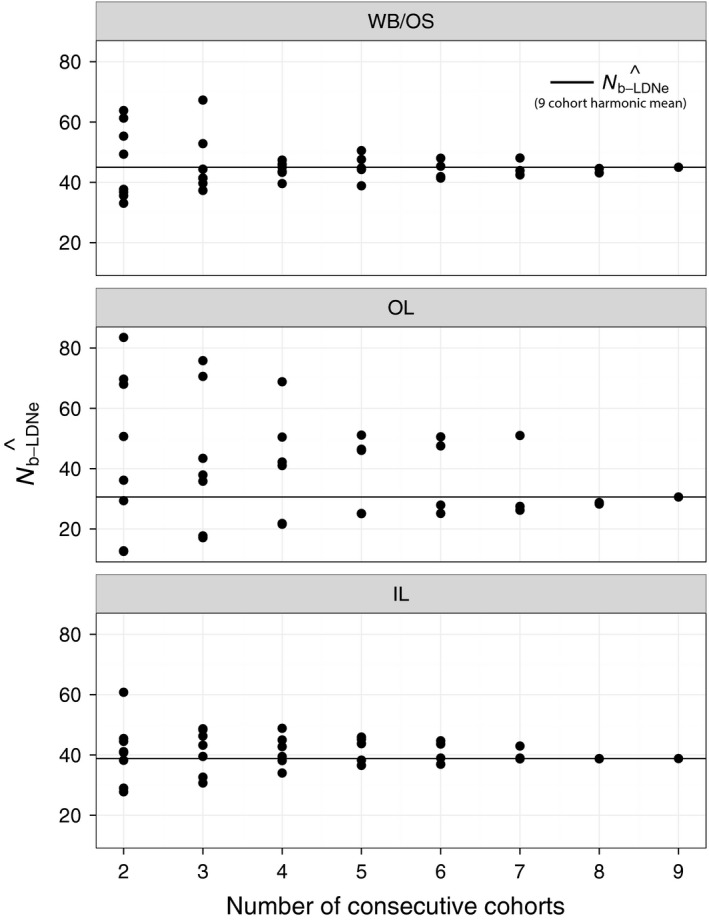
Test of the number of consecutive cohorts needed to reliably estimate ${\hat{N}}_{b-\mathrm{LDNe}}$ for each of the three brook trout subpopulations. The *x*‐axis represents the number of consecutive cohorts subsampled (from two successive cohorts to nine). The *y*‐axis shows the harmonic mean of the ${\hat{N}}_{b-\mathrm{LDNe}}$ obtained from each of the successive cohorts subsampled. All possible successive subsets for each value of consecutive cohorts were obtained. For example, there were eight possible consecutive cohorts of size two for the nine cohorts. The harmonic mean of all nine cohorts is shown with a horizontal line. This value must equal the value of consecutive cohorts = 9, by definition, and is shown for heuristic purpose

**Figure 9:**
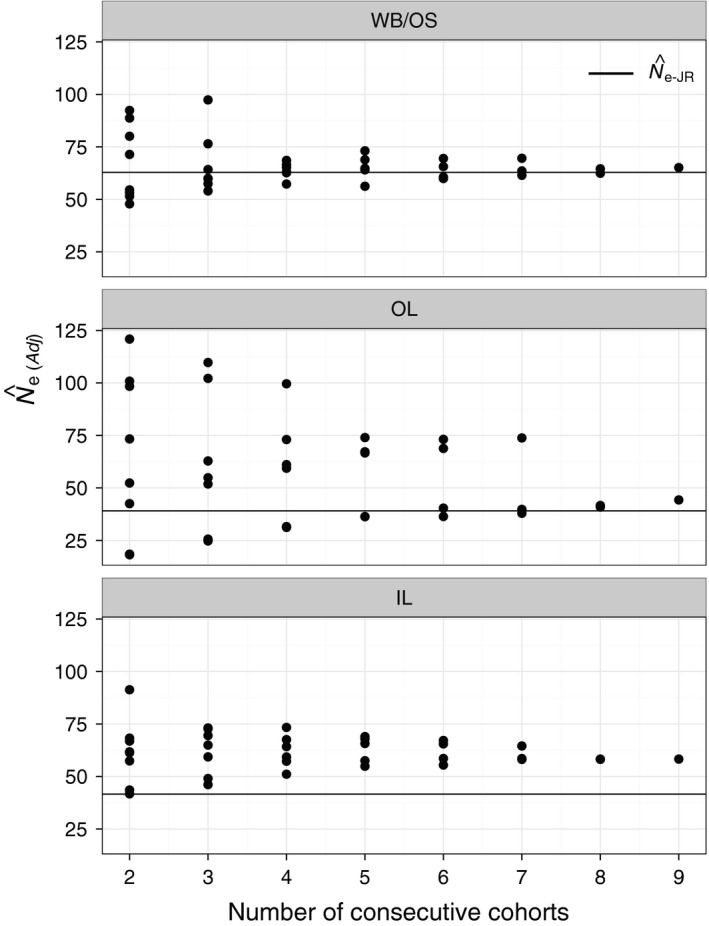
Test of the number of consecutive cohorts needed to reliably estimate ${\hat{N}}_{e(\mathrm{Adj})}$ from ${\hat{N}}_{b-\mathrm{LDNe}}$ for each of the three brook trout subpopulations. The *x*‐axis represents the number of consecutive cohorts subsampled (from two successive cohorts to nine). The *y*‐axis shows ${\hat{N}}_{e}$ following the Waples et al. (2014) approach. The harmonic means of the ${\hat{N}}_{b-\mathrm{LDNe}}$ obtained from subsampled successive cohorts were used to obtain ${\hat{N}}_{e(\mathrm{Adj})}$. All possible successive subsets for each value of consecutive cohorts were obtained. Overall ${\hat{N}}_{e-\mathrm{JR}}$ obtained with the Jorde and Ryman (Jorde and Ryman (1995) approach is shown with a horizontal line

Further analysis of subsets of consecutive cohorts provided guidelines for the approximate number of cohorts needed to reliably estimate harmonic mean ${\hat{N}}_{b-\mathrm{LDNe}}$ for a population and subsequently for the number of cohorts that might be needed to reliably apply the Waples et al. (2014) approach. Harmonic mean ${\hat{N}}_{b-\mathrm{LDNe}}$ from approximately four or five consecutive cohorts began to converge on the overall harmonic mean from all nine cohorts in WB/OS and IL (Figure 8). In OL, the subpopulation with greater temporal variation in ${\hat{N}}_{b-\mathrm{LDNe}}$, eight consecutive cohorts were required for a similar amount of convergence. Patterns for ${\hat{N}}_{e(\mathrm{Adj})}$ were identical (Figure 9) as these values were calculated directly from the same subsets of cohorts used to obtain estimates of ${\hat{N}}_{b-\mathrm{LDNe}}$ shown in Figure 8. The tendency for ${\hat{N}}_{e(\mathrm{Adj})}$ to be greater than ${\hat{N}}_{e-\mathrm{JR}}$ in OL and IL was apparent in this analysis (Figure 9).

## 4 Discussion

We examined patterns of subpopulation‐specific *N*_b_ over time and the temporal and spatial scales to which estimates of ${\hat{N}}_{b-\mathrm{LDNe}}$ apply in an exhaustively sampled and demographically well‐understood stream‐resident brook trout metapopulation. ${\hat{N}}_{b-\mathrm{LDNe}}$ from each subpopulation appeared to correspond to the local (subpopulation) spatial scale and a cohort‐specific temporal scale. There was little correlation in ${\hat{N}}_{b-\mathrm{LDNe}}$ among sites, there was a strong correlation between ${\hat{N}}_{b-\mathrm{LDNe}}$ and measures of family structure, and ${\hat{N}}_{b-\mathrm{LDNe}}$ exhibited a quadratic relationship with autumn stream flow in WB/OS and IL and a positive linear relationship with flow in OL. Upon pooling genetically divergent populations, ${\hat{N}}_{b-\mathrm{LDNe}}$ exhibited mixture LD and did not appear to approach an (*albeit* unknown) global or metapopulation ${\hat{N}}_{b}$. Estimation of generational ${\hat{N}}_{e}$ (${\hat{N}}_{e(\mathrm{Adj})}$) from the harmonic mean of cohort‐specific ${\hat{N}}_{b}$ was reasonably close to estimates of ${\hat{N}}_{e-\mathrm{JR}}$. Our results further suggest that ${\hat{N}}_{b}$ from at least four consecutive cohorts might be needed to reliably estimate harmonic mean ${\hat{N}}_{b}$ for a population or subpopulation.

### 4.1 Population genetic structure

It is important to estimate population genetic structure prior to estimation of effective population size (Ryman, Allendorf, Jorde, Laikre, & Hossjer, 2014). We found strong evidence for three subpopulations. Genetic differentiation was absent between WB and OS. OS appeared to be an extension of habitat in WB, which is likely used for spawning and early rearing. A perched culvert was in place at the mouth of OS during our study. We previously demonstrated that this culvert served as a partial barrier to fish movement (Kanno et al., 2014). WB fish intermittently accessed OS and at times had very high reproductive success (Kanno et al., 2014). It is possible that a small resident component remained in OS but any genetic divergence that might have developed between WB and OS during years with little to no access through the perched culvert must have been swamped during years where fish from the WB reproduced in OS.

There was evidence for genetic divergence between OL and WB/OS. Yet, we also found evidence for a substantial amount of movement of fish into OL and from OL to WB, even prior to the first time young‐of‐year were sampled in any given cohort (between hatching in the spring and summer or autumn sampling). Our genetically based estimates of movement were similar to previous demographic modeling results, but with elevated estimates of movement from WB/OS to OL (Letcher et al., 2014). The level of genetic differentiation observed (*F*_ST_ = 0.06, $F_{\mathrm{ST}}^{\prime }$ = 0.16) would require a combination of (i) substantial nonreproductive movement among subpopulations at various life stages, (ii) natal homing, and (iii) reduced reproductive success of non‐natal fish in each location. We have previously reported evidence of movement related to enhanced survival and growth in the connected subpopulations (Kanno et al., 2014; Letcher et al., 2014). Evidence for homing and reduced reproductive success of non‐natal individuals comes from a variety of salmonids (Hendry, 2000; Hendry & Strearns, 2004; Mortensen, Wertheimer, Maselko, & Taylor, 2002; Wood, 1995).

IL was genetically divergent from the remainder of the subpopulations, as expected from past work (Letcher et al., 2007). We observed a small signal of admixture from WB/OS into IL with the STRUCTURE analyses, which was unexpected based on past tagging studies that included a PIT tag antenna located immediately upstream of the 2.3‐m waterfall at the mouth of this stream. This signal of upstream movement into IL might reflect limits of the STRUCTURE model with 12 microsatellites. Letcher et al. (2007) previously reported a life history shift toward the demographic importance of smaller fish in IL compared to the other subpopulations. Emigration from IL to WB/OS (mean for all individual analysis = 0.05) and OL (mean for all individual analysis = 0.02) provides an opportunity for future tests of local adaptation.

Limitations of the STRUCTURE model when sampling is uneven must also be acknowledged (Puechmaille 2016). Small sample size in OS might have biased our results and caused WB to be merged with OS. For the sibling‐purged STRUCTURE analysis used to infer *K*, mean sample size across cohorts for WB was 98.9, for OS was 12.3, for OL was 25.4, and for IL was 18.0. Thus, any downward bias associated with uneven sampling did not influence our interpretations regarding OL and IL. It remains possible that we have artificially merged WB and OS. However, we would have expected a greater signal of genetic differentiation between WB and OS when sample sizes were larger in OS (e.g., sibling‐purged sample size in the 2007 cohort was greater in OS than OL and IL), and this was not the case. Furthermore, models based on *K *=* *4 did not split WB from OS; rather, small proportions of the genome of individuals were assigned to a fourth group that was not associated with a specific geographic location or cohort. Merging WB and OS appears to be the most biologically defensible interpretation of our data.

### 4.2 ${\hat{N}}_{b}$ and ${\hat{N}}_{C}$ over time within subpopulations

Harmonic means of point estimates of ${\hat{N}}_{b-\mathrm{LDNe}}$ across all cohorts were greatest in WB/OS, intermediate in IL, and lowest in OL. Larger effective population size estimates from WB/OS were mainly driven by individuals from the mainstem (WB). WB contained members of on average 75% (range 62%–91%) of all of the full‐sib families detected in a given cohort. The overwhelming influence of WB on ${\hat{N}}_{b-\mathrm{LDNe}-\mathrm{WB}/\mathrm{OS}}$ underlines the importance of the mainstem for reproduction and/or early rearing in this study system. This extends past analyses that have focused on the importance of tributary habitat for spawning and early rearing in this stream network (Kanno et al., 2014; Letcher et al., 2007, 2014).

IL had larger mean estimates of ${\hat{N}}_{b-\mathrm{LDNe}}$ than OL. These streams have approximately the same amount of habitat. Reproduction at a smaller body size in IL compared to OL (Letcher et al., 2007) for this species that exhibits size‐based fecundity provides a possible explanation for larger *N*_b_ estimates in IL compared to OL. Across cohorts, mean ${\hat{N}}_{\mathrm{fam}}$ was similar in IL (29.7) as OL (30.9) but mean $\hat{\text{FE}}$ was greater in IL (0.929) than OL (0.885). Production of a similar number of families but lower reproductive variance (greater family evenness) in IL compared to OL is consistent with higher *N*_b_ and *N*_e_ in IL.

${\hat{N}}_{b-\mathrm{LDNe}}$ were relatively stable despite declines in abundance in each subpopulation. This relative stability in ${\hat{N}}_{b-\mathrm{LDNe}}$ despite large variance in ${\hat{N}}_{C}$ led to substantial variation in ${\hat{N}}_{b-\mathrm{LDNe}}/{\hat{N}}_{C}$ ratios over time. ${\hat{N}}_{b-\mathrm{LDNe}}/{\hat{N}}_{C}$ varied almost 12‐fold (range = 0.08–0.92) across the three subpopulations. We previously tested the genetic compensation hypothesis and did not find evidence that variance in reproductive success was lower at reduced abundance in this system (Whiteley et al., 2015). Instead, the weight of evidence supported the hypothesis that ${\hat{N}}_{b}$ is determined by amount and quality of reproductive and early rearing habitat. Here, we extend the observation of relatively stable ${\hat{N}}_{b}$ compared to the high temporal variance in ${\hat{N}}_{C}$ within each of the subpopulations in this system of populations.

High variance in ${\hat{N}}_{b}/{\hat{N}}_{C}$ has been observed in other studies (Palstra & Fraser, 2012; Ruzzante et al., 2016). Palstra and Fraser (2012) found a median ${\hat{N}}_{b}/{\hat{N}}_{C}$ value of 0.23 across 62 estimates that properly linked ${\hat{N}}_{b}$ and ${\hat{N}}_{C}$. High variability in the ${\hat{N}}_{b}/{\hat{N}}_{C}$ ratio over time suggests that ${\hat{N}}_{b}$ does not consistently follow any trend in ${\hat{N}}_{C}$ and might not be useful for detecting subtle population trend as a genetic monitoring metric, although it remains possible that ${\hat{N}}_{b}$ could be used to monitor large change in abundance (Whiteley et al., 2015).

There are several caveats worth mentioning with respect to *N*_b_ estimation in this study. First, we used STRUCTURE to define subpopulations and then estimated ${\hat{N}}_{b}$ with LD‐based estimator. The STRUCTURE‐defined subpopulations should minimize HW and linkage disequilibrium within subpopulations (Pritchard et al., 2000). Minimizing LD could cause an upward bias in the subpopulation‐specific ${\hat{N}}_{b}$ presented here. However, we defined subpopulations with STRUCTURE originally based on a smaller subset of the data (YOY‐only) with one randomly selected full‐sib per family. We then used this value of *K* and reran STRUCTURE with all individuals in our data set. Any minimization of LD within subpopulations that occurred in the analysis of the YOY‐only data set should be overwhelmed by the LD present in the entire data set due to cohort‐specific family structure. Second, we used a random mating model with the LDNe program. We previously (Whiteley et al., 2015) used the monogamy mating model in LDNe because brook trout appear to conform more closely to monogamy than random mating (Coombs, 2010). The monogamy mating model effectively doubles the estimates of ${\hat{N}}_{b-\mathrm{LDNe}}$ compared to the random mating model. We needed to use the random mating model here to allow comparison of ${\hat{N}}_{e}$ from the Waples et al. (2014) approach to ${\hat{N}}_{e-\mathrm{JR}}$ obtained from the Jorde and Ryman approach, which assumes random mating (Jorde & Ryman, 1995). The mating model we used will not influence relative comparison of ${\hat{N}}_{b-\mathrm{LDNe}}$ over time (within or across subpopulations), unless degree of polygamy also varies over time (Whiteley et al., 2015). Further, relationships we observed between ${\hat{N}}_{b}$ and stream flow would likely have been stronger if brook trout exhibit a density‐dependent increase in polygamy within these subpopulations (Whiteley et al., 2015).

### 4.3 Do *N*_b_ estimates from subpopulations within a metapopulation apply to a local (subpopulation) scale and cohort‐specific timescale?

Our results strongly suggest that cohort‐specific ${\hat{N}}_{b-\mathrm{LDNe}}$ corresponded to the subpopulation spatial scale and temporally to the cohort upon which estimates are based. ${\hat{N}}_{b-\mathrm{LDNe}}$exhibited weak temporal synchrony across subpopulations, strong relationships between cohort‐specific measures of family structure, and significant relationships with stream flow, particularly in WB/OS and OL. We expected more temporal synchrony across subpopulations in ${\hat{N}}_{b-\mathrm{LDNe}}$ if the signal applied to a spatial scale larger than the subpopulation. We also expected the localized measures of the number of full‐sib families and the variance in the size of those families to show little relationship with subpopulation ${\hat{N}}_{b-\mathrm{LDNe}}$, if ${\hat{N}}_{b-\mathrm{LDNe}}$ applied to a larger spatial scale.

The relationship with autumn stream flow in each subpopulation was also consistent with localized environmental effects on *N*_b_. We previously reported a significant quadratic relationship between autumn flow and ${\hat{N}}_{b-\mathrm{LDNe}}$ for combined ${\hat{N}}_{b-\mathrm{LDNe}-\mathrm{WB}/\mathrm{OS}/\mathrm{OL}}$ over the same time period (Whiteley et al., 2015). In this previous analysis, we did not account for substructure among these three streams. The relationship reported here was slightly weaker (*R* ^2^ = .66 compared to *R* ^2^ = .73 previously), largely because ${\hat{N}}_{b-\mathrm{LDNe}-\mathrm{OL}}$ in 2008 was so low that it reduced ${\hat{N}}_{b-\mathrm{LDNe}-\mathrm{WB}/\mathrm{OS}/\mathrm{OL}}$ and this was a highly influential point in the regression. We previously discussed hypotheses for a quadratic relationship between ${\hat{N}}_{b-\mathrm{LDNe}}$ and autumn flow, including increased competition and limitations on available spawning habitat during reproduction at low flows and reduced habitat quality (including redd scouring) at high flows (Whiteley et al., 2015). The linear relationship in OL is consistent with increased competition at low flows without the reduction in *N*_b_ at high flows. It is possible we did not see reduced ${\hat{N}}_{b-\mathrm{LDNe}-\mathrm{OL}}$ at higher flows because of the lower range of flows experienced by OL compared to WB/OS. However, we found evidence (*albeit* nonsignificant) for a quadratic relationship in IL, which suggests that smaller drainages might also experience reduced *N*_b_ at relatively high flows. These results provide further support for the hypothesis that ${\hat{N}}_{b-\mathrm{LDNe}}$ are related to quantity and quality of spawning and early rearing habitat (Whiteley et al., 2015).

Our work provides empirical support that estimates of ${\hat{N}}_{b-\mathrm{LDNe}}$ provide local (subpopulation) ${\hat{N}}_{b}$ in the face of gene flow in brook trout populations. ${\hat{N}}_{b-\mathrm{LDNe}}$ were robust to admixture, where the estimated proportion of individuals with admixture within subpopulations and cohorts ranged from 0 to 0.33. Removing admixed individuals had a weak effect in predicted direction (estimate increased once admixed individuals removed) in 15% of cohort‐specific ${\hat{N}}_{b-\mathrm{LDNe}}$ estimates. We chose to focus specifically on the effects of admixture after removing the effects of gene flow on effective size estimates, that is, we assigned individuals to putative natal populations prior to conducting estimates with and without admixed individuals. We might have seen a larger influence on estimates had we included putative migrants in our samples. However, both Gomez‐Uchida et al. (2013) and Serbezov et al. (2012b) found little influence of migrants on estimates of *N*_b_ or *N*_e_ when estimates included or excluded putative migrants. These empirical results are consistent with simulation‐based results of Waples and England (2011) that suggest estimates of local *N*_e_ are robust to equilibrium levels of gene flow between 5% and 10%. Our work also reveals that the effect of gene flow on estimates on ${\hat{N}}_{b-\mathrm{LDNe}}$ varies over time, and in some cohorts, gene flow could cause bias in estimates that would be difficult to discern in analyses based on single cohorts.

Our results suggest that mixture LD is likely to prevent LD estimators from providing estimates of metapopulation *N*_b_ and provide insight into the degree of genetic divergence that might be expected to cause mixture LD in natural populations. Previous results based on island model simulations found that equilibrium migration that is rare and episodic can occasionally lead to mixture LD (Waples & England, 2011). Waples and England (2011) also found that nonequilibrium pulse migration of strongly divergent individuals can create strong mixture LD. Here, harmonic mean ${\hat{N}}_{b-\mathrm{LDNe}}$ decreased as divergent subpopulations were pooled. When we added IL individuals to WB/OS (whether OL was present or not), ${\hat{N}}_{b-\mathrm{LDNe}}$ was reduced. Genetic differentiation was only slightly greater between WB/OS and IL (*F*_ST_ = 0.09, $F_{\mathrm{ST}}^{\prime }$ = 0.20) than between WB/OS and OL (*F*_ST_ = 0.06, $F_{\mathrm{ST}}^{\prime }$ = 0.16). Importantly, even though we were drawing from a larger pool of parents as we pooled subpopulations (${\hat{N}}_{\mathrm{fam}}$increased 17% for WB/OS/IL compared to WB/OS), which should have elevated ${\hat{N}}_{b-\mathrm{LDNe}}$ as the spatial scale increased, mixture LD more than negated this effect. In the case of the addition of OL to WB/OS (${\hat{N}}_{\mathrm{fam}}$ increased 21% for WB/OS/OL compared to WB/OS), the effect of mixture LD appeared to cancel out (without depressing) the effects of increasing the pool of possible parents on ${\hat{N}}_{b-\mathrm{LDNe}}$. Our results are similar to a study of Atlantic salmon (*Salmo salar*) that found that pooling samples with *F*_ST_ < 0.005 did not depress ${\hat{N}}_{e}$, but pooling samples with *F*_ST_ of approximately 0.05 did (Palstra & Ruzzante, 2011). Similarly, in a comparison of three salmonid species in the same landscape, Gomez‐Uchida et al. (2013) found evidence of greater suppression of pooled ${\hat{N}}_{e}$ for the species with the most population genetic differentiation.

### 4.4 Generational ${\hat{N}}_{e}$

We provide an empirical examination of the relationship between ${\hat{N}}_{e(\mathrm{Adj})}$ (Waples et al., 2014) and ${\hat{N}}_{e-\mathrm{JR}}$ (Jorde & Ryman, 1995), two approaches to obtain generational ${\hat{N}}_{e}$ for an iteroparous organism with age structure. Our empirical demonstration is based on an organism with an *N*_b_/*N*_e_ ratio in the range of 0.5–0.7, depending on assumptions made about variance in reproductive success of same‐age, same‐sex individuals. ${\hat{N}}_{e(\mathrm{Adj})}$ was greater than empirical estimates of ${\hat{N}}_{e-\mathrm{JR}}$ by 7%–50%. ${\hat{N}}_{e(\mathrm{Adj})}$ and ${\hat{N}}_{e-\mathrm{JR}}$ were more similar in WB/OS and OL than in IL. The greatest congruence occurred in WB/OS, the largest system with the greatest genetic diversity and least intercohort genetic drift. The greatest divergence between ${\hat{N}}_{e(\mathrm{Adj})}$ and ${\hat{N}}_{e-\mathrm{JR}}$ occurred in IL, the isolated subpopulation with the lowest genetic diversity and most intercohort genetic drift. It is not possible to discern whether these two generational *N*_e_ estimators are most similar by chance in WB/OS and OL or if bias caused their divergence in IL. One explanation for the greatest discrepancy between the estimators in IL is inaccuracy in either or both due to low genetic diversity in IL, despite a large sample size relative to the likely true effective size (mean *S* across cohorts was 146). It is also possible that violations of the simplifying assumptions of constant population size and stable age structure (Waples et al., 2013) in the AgeNe approach used to estimate ${\hat{N}}_{e(\mathrm{Adj})}$ had subpopulation‐specific effects.

Subpopulation‐specific violations of assumptions of the Jorde and Ryman (1995) approach used to estimate ${\hat{N}}_{e-\mathrm{JR}}$ should also be considered. The Jorde and Ryman estimator has been shown to be minimally biased when used on multiple consecutive cohorts for organism with overlapping generations (Charlier et al., 2012), as was the case with our data set. In addition, our life table parameters should be robust because they were based on extensive individual tagging data collected seasonally (4× per year) throughout the study period (Bassar et al., 2016; Letcher et al., 2014). However, the Jorde and Ryman estimator assumes stable abundance (Jorde & Ryman, 1995), which was not the case here. Declining abundance could cause allele frequency variation among cohorts to be elevated relative to a stable population. This would serve to elevate estimates of $F_{S}^{\prime }$ relative to a stable population and to depress ${\hat{N}}_{e-\mathrm{JR}}$. The isolated subpopulation (IL) exhibited the greatest signal of intercohort drift and WB/OS the least, but $F_{S}^{\prime }$ was only slightly greater for IL (0.037) compared to OL (0.035). Thus, the effect of drift might explain why ${\hat{N}}_{e-\mathrm{JR}}$ was lower than ${\hat{N}}_{e(\mathrm{Adj})}$ for IL and OL relative to WB/OS, but by itself does not fully explain why IL showed the greatest discrepancy between generational *N*_e_ estimates. Other parameters used by the Jorde–Ryman approach, including life table parameters and the correction factor for overlapping generations (*C*), appear to be minimally influenced by demographic fluctuations (Jorde & Ryman, 1995). Recent work also demonstrates that gene flow can cause considerable downward bias in estimates of local (subpopulation) ${\hat{N}}_{e}$ obtained from the temporal method (Ryman et al., 2014). We have comprehensively sampled brook trout throughout our study area. Brook trout occur upstream of the study reaches in WB, OL, and IL and downstream of the study reach in WB. It is possible that small amounts of gene flow from outside our study area have caused us to underestimate ${\hat{N}}_{e-\mathrm{JR}}$ and the effect could be subpopulation‐specific, but testing this possibility would require further analyses.

Our study suggests that estimates of ${\hat{N}}_{b}$ based on multiple successive cohorts might be needed to reliably estimate harmonic mean ${\hat{N}}_{b}$ for a population. This is an important consideration for attempts to characterize ${\hat{N}}_{b}$ for a population from limited data. ${\hat{N}}_{b}$ is influenced by many factors, including variable age at maturity and competitive interactions during reproduction and early survival, which can be environmentally mediated (Whiteley et al., 2015). A harmonic mean based on data from one or a few years might not adequately capture among‐cohort variability. We found that at least four successive cohorts appear to be needed for subpopulations with less variation in ${\hat{N}}_{b}$. More successive cohorts were needed for the subpopulation with the greatest temporal variation in ${\hat{N}}_{b}$ (OL). It follows that estimation of ${\hat{N}}_{e(\mathrm{Adj})}$ based on estimates of harmonic mean ${\hat{N}}_{b}$ would also be susceptible to this sampling effect.

## 5 Conclusions

Our results regarding the spatial scale of inference for *N*_b_ should apply broadly to many taxa that exhibit overlapping generations and metapopulation structure. Our results also suggest that linking measures of *N*_b_ to environmental covariates will best be achieved through cohort‐specific estimates of *N*_b_. It remains to be seen whether ${\hat{N}}_{b}$ in other locations and taxa tend to vary as much as they do in the populations examined here, where cohort‐specific variation in family structure is pronounced. For example, the range in the largest full‐sib family size estimated across the nine cohorts was 10–71 (Whiteley et al., 2015). It will also be challenging to link environmental variation to cohort‐specific ${\hat{N}}_{b}$ if ${\hat{N}}_{b}$ is large, simply because of the extra challenges presented in estimating large values of this measure (Tallmon et al., 2010). It is possible that new approaches that harness many SNPs and linkage relationships will help to overcome this challenge.

We have previously defined effective sampling strategies for cohort‐specific ${\hat{N}}_{b}$ within subpopulations (Whiteley et al., 2012). If metapopulation structure is likely, we recommend that researchers err on the side of overly spatially inclusive samples. If genetically differentiated subpopulations are included, they can later be screened prior to *N*_b_ or *N*_e_ estimation by the implementation of standard population genetic approaches to detect population substructure.

Finally, lack of temporal synchrony in ${\hat{N}}_{b-\mathrm{LDNe}}$ across nearby subpopulations that exchange migrants observed here provides insight into maintenance of genetic diversity within metapopulations. In some breeding seasons where the dominant subpopulation faltered, less productive subpopulations had higher ${\hat{N}}_{b}$. For example, when WB/OS was at its lowest value of ${\hat{N}}_{b-\mathrm{LDNe}}$ (2006 cohort), OL was at its highest value. Only one year (2002) had consistently low ${\hat{N}}_{b-\mathrm{LDNe}}$ across all three subpopulations. This buffering of effective genetic contribution across subpopulations could play an important role in maintaining genetic diversity in and ultimately aid persistence of connected sets of populations and could be an additional, but previously unconsidered, component of the portfolio effect (Schindler et al., 2010).

## Data Archiving Statement

Data files with individual information (sample date and location, cohort, body size, full‐sib family ID, and microsatellite genotypes) are available from DRYAD (https://doi.org/10.5061/dryad.s4td3).
