# Supplementary material for: Keeping things local: Subpopulation N b and N e in a stream network with partial barriers to fish migration
Source: Evol Appl. 2017 Feb 9;10(4):348–65. doi: 10.1111/eva.12454 (PMC5367083; doi:10.1111/eva.12454)
Supplement: Supplementary file 1 [file EVA-10-348-s001.docx]

Table S1. Number of alleles per locus and per cohort for each of the three STRUCTURE-defined brook trout subpopulations in the WB metapopulation.

| Cohort | *Sfo*-*C113* | *Sfo*-*C88* | *Sfo*-*D75* | *Sfo*-*D100* | *Sfo*-*C24* | *Sfo*-*C115* | *Sfo*-*C129* | *Ssa*-*D237* | *Sfo*-*C38* | *Sfo*-*C86* | *Sfo*-*B52* | *Sfo*-*D91a* |
| --- | --- | --- | --- | --- | --- | --- | --- | --- | --- | --- | --- | --- |
| WB/OS |  |  |  |  |  |  |  |  |  |  |  |  |
| 2001 | 8 | 4 | 7 | 7 | 4 | 8 | 5 | 17 | 5 | 5 | 7 | 3 |
| 2002 | 10 | 5 | 8 | 12 | 4 | 15 | 5 | 31 | 8 | 5 | 10 | 4 |
| 2003 | 8 | 3 | 4 | 7 | 1 | 3 | 2 | 9 | 3 | 2 | 5 | 3 |
| 2004 | 7 | 4 | 5 | 7 | 4 | 6 | 5 | 13 | 5 | 5 | 8 | 3 |
| 2005 | 11 | 5 | 7 | 11 | 4 | 16 | 6 | 29 | 8 | 5 | 11 | 3 |
| 2006 | 6 | 3 | 4 | 7 | 1 | 2 | 2 | 7 | 3 | 2 | 4 | 3 |
| 2007 | 7 | 4 | 5 | 8 | 4 | 6 | 5 | 19 | 6 | 5 | 6 | 4 |
| 2008 | 10 | 6 | 8 | 12 | 4 | 14 | 6 | 28 | 8 | 6 | 11 | 3 |
| 2009 | 7 | 3 | 4 | 8 | 1 | 5 | 2 | 8 | 3 | 2 | 4 | 3 |
| mean | 8.2 | 4.1 | 5.8 | 8.8 | 3.0 | 8.3 | 4.2 | 17.9 | 5.4 | 4.1 | 7.3 | 3.2 |
| OL |  |  |  |  |  |  |  |  |  |  |  |  |
| 2001 | 8 | 3 | 5 | 7 | 4 | 7 | 5 | 18 | 7 | 4 | 8 | 3 |
| 2002 | 9 | 5 | 8 | 12 | 4 | 12 | 8 | 27 | 9 | 6 | 10 | 4 |
| 2003 | 6 | 3 | 5 | 7 | 1 | 5 | 2 | 9 | 3 | 2 | 4 | 3 |
| 2004 | 7 | 3 | 6 | 7 | 4 | 7 | 5 | 18 | 6 | 6 | 7 | 4 |
| 2005 | 7 | 6 | 7 | 14 | 4 | 13 | 5 | 30 | 9 | 5 | 11 | 4 |
| 2006 | 7 | 3 | 4 | 8 | 2 | 3 | 2 | 7 | 3 | 3 | 4 | 3 |
| 2007 | 6 | 4 | 5 | 7 | 4 | 4 | 5 | 15 | 6 | 5 | 8 | 3 |
| 2008 | 10 | 5 | 8 | 11 | 4 | 7 | 5 | 23 | 8 | 5 | 9 | 3 |
| 2009 | 6 | 3 | 4 | 7 | 2 | 4 | 2 | 9 | 3 | 2 | 4 | 3 |
| mean | 7.3 | 3.9 | 5.8 | 8.9 | 3.2 | 6.9 | 4.3 | 17.3 | 6.0 | 4.2 | 7.2 | 3.3 |
| IL |  |  |  |  |  |  |  |  |  |  |  |  |
| 2001 | 9 | 3 | 5 | 8 | 4 | 6 | 5 | 15 | 6 | 4 | 7 | 4 |
| 2002 | 8 | 5 | 6 | 12 | 4 | 8 | 7 | 27 | 9 | 4 | 10 | 4 |
| 2003 | 9 | 4 | 5 | 6 | 1 | 4 | 2 | 11 | 3 | 2 | 4 | 3 |
| 2004 | 6 | 3 | 3 | 7 | 3 | 8 | 4 | 15 | 5 | 5 | 6 | 4 |
| 2005 | 11 | 8 | 9 | 12 | 4 | 15 | 6 | 29 | 8 | 5 | 9 | 4 |
| 2006 | 5 | 3 | 4 | 6 | 1 | 3 | 4 | 7 | 3 | 2 | 3 | 3 |
| 2007 | 9 | 4 | 8 | 7 | 4 | 8 | 5 | 14 | 6 | 5 | 8 | 3 |
| 2008 | 10 | 5 | 8 | 13 | 4 | 15 | 6 | 27 | 9 | 5 | 13 | 3 |
| 2009 | 6 | 3 | 6 | 7 | 2 | 5 | 2 | 9 | 3 | 5 | 4 | 3 |
| mean | 8.1 | 4.2 | 6.0 | 8.7 | 3.0 | 8.0 | 4.6 | 17.1 | 5.8 | 4.1 | 7.1 | 3.4 |

Table S2. Pairwise *F*_ST_ (Nei’s *G*_ST_; above diagonal) and pairwise *F’*_ST_ (*G’’*_ST_, below diagonal) for three subpopulations (OL, WB/OS, and IL) with the West Brook study system. Estimates are based on entire cohorts following structure assignments to putative natal locations for each individual. Sample size is shown above the matrix for each cohort and corresponds to the subpopulation in a given column.

| 2001 | 251 | 636 | 239 |
| --- | --- | --- | --- |
|  | OL | WB/OS | IL |
| OL | -- | 0.06 | 0.21 |
| WB/OS | 0.16 | -- | 0.11 |
| IL | 0.39 | 0.23 | -- |
|  |  |  |  |
| 2002 | 113 | 484 | 224 |
|  | OL | WB/OS | IL |
| OL | -- | 0.08 | 0.18 |
| WB/OS | 0.20 | -- | 0.10 |
| IL | 0.35 | 0.22 | -- |
|  |  |  |  |
| 2003 | 149 | 734 | 278 |
|  | OL | WB/OS | IL |
| OL | -- | 0.05 | 0.19 |
| WB/OS | 0.12 | -- | 0.11 |
| IL | 0.37 | 0.24 | -- |
|  |  |  |  |
| 2004 | 199 | 587 | 148 |
|  | OL | WB/OS | IL |
| OL | -- | 0.06 | 0.18 |
| WB/OS | 0.15 | -- | 0.09 |
| IL | 0.35 | 0.20 | -- |
|  |  |  |  |
| 2005 | 129 | 431 | 77 |
|  | OL | WB/OS | IL |
| OL | -- | 0.06 | 0.20 |
| WB/OS | 0.15 | -- | 0.09 |
| IL | 0.38 | 0.19 | -- |

| 2006 | 60 | 352 | 53 |
| --- | --- | --- | --- |
|  | OL | WB/OS | IL |
| OL | -- | 0.09 | 0.19 |
| WB/OS | 0.22 | -- | 0.08 |
| IL | 0.38 | 0.17 | -- |
|  |  |  |  |
| 2007 | 91 | 186 | 76 |
|  | OL | WB/OS | IL |
| OL | -- | 0.06 | 0.14 |
| WB/OS | 0.13 | -- | 0.07 |
| IL | 0.28 | 0.17 | -- |
|  |  |  |  |
| 2008 | 121 | 379 | 67 |
|  | OL | WB/OS | IL |
| OL | -- | 0.05 | 0.18 |
| WB/OS | 0.14 | -- | 0.11 |
| IL | 0.37 | 0.23 | -- |
|  |  |  |  |
|  |  |  |  |
| 2009 | 147 | 567 | 154 |
|  | OL | WB/OS | IL |
| OL | -- | 0.06 | 0.16 |
| WB/OS | 0.14 | -- | 0.08 |
| IL | 0.32 | 0.18 | -- |

Table S3. Life table data for brook trout from West Brook. Demographic rates were empirically derived separately for WB/OS and OL and for IL. These data were used as input for program AgeNe to determine the ratio of *N*_b_ to *N*_e_ and with the program GONe to implement the Jorde and Ryman approach to estimate *N*_e_.

| Age | Annual Survival (95% CI) | Birth | Birth Lower Range | Birth Upper Range |
| --- | --- | --- | --- | --- |
| *WB/OS and OL* |  |  |  |  |
| 1 | 0.253 (0.236-0.271) | 0.971 | 0.00 | 1.92 |
| 2 | 0.145 (0.135-0.156) | 29.86 | 16.45 | 41.91 |
| 3 | 0.177 (0.156-0.199) | 120.38 | 95.09 | 157.53 |
| 4 | 0.083 (0.044-0.137) | 240.53 | 159.62 | 362.47 |
| 5 | 0.056 (0.005-0.208) | 314.46 | 217.02 | 455.65 |
| *IL* |  |  |  |  |
| 1 | 0.366 (0.315-0.416) | 0.06 | 0.00 | 0.200 |
| 2 | 0.231 (0.203-0.260) | 26.30 | 16.45 | 35.70 |
| 3 | 0.184 (0.149-0.222) | 79.36 | 95.09 | 97.62 |
| 4 | 0.109 (0.048-0.198) | 120.38 | 104.53 | 138.64 |
| 5 | 0.030 (0.000-0.244) | 147.55 | 107.20 | 203.10 |

Table S4. Effect of LD from admixture on $\hat{N}_{b-LDNe}$ and family structure summary statistics. Cohorts are defined by the year of emergence. Each variable is listed with all individuals assigned to one of the three natal subpopulations, and with admixed (0.3 < *q* < 0.7) individuals removed (NA - no admixed; Diff. columns). *N*_G_ is the number of individuals genotyped per cohort. Admixture is the proportion of fish from a cohort/population combination that had *q*-values between 0.3 and 0.7, based on analysis of the entire cohorts. $\hat{N}_{b-LDNe}$ (point estimates only) is the effective number of breeders.$\hat{FE}$ is family evenness, a measure inversely related to variance in the full-sib family distribution of each cohort. $\hat{N}_{fam}$ is the number of estimated full-sib families. The difference between estimates without (no admixed) and with admixed individuals is shown following $\hat{N}_{b}$, $\hat{FE}$, and $\hat{N}_{fam}$. If the inclusion of admixed individuals caused estimates of $\hat{N}_{b-LDNe}$ to be biased low, we predicted that this difference would be positive ($\hat{N}_{b-LDNe}$(NA) > $\hat{N}_{b-LDNe}$).

| Cohort | *N*_G_ | *N*_G_  (NA) | Admixture | $\hat{N}_{b-LDNe}$ | $\hat{N}_{b}$(NA) | Diff. | $\hat{FE}$ | $\hat{FE}$ (NA) | Diff. | $\hat{N}_{fam}$ | $\hat{N}_{fam}$ (NA) | Diff. |
| --- | --- | --- | --- | --- | --- | --- | --- | --- | --- | --- | --- | --- |
| *WB/OS* |  |  |  |  |  |  |  |  |  |  |  |  |
| 2001 | 636 | 567 | 0.08 | 68.8 | 65.0 | -3.8 | 0.910 | 0.909 | -0.001 | 202 | 183 | -19 |
| 2002 | 484 | 426 | 0.10 | 25.1 | 22.9 | -2.2 | 0.856 | 0.848 | -0.008 | 114 | 105 | -9 |
| 2003 | 734 | 633 | 0.14 | 75.5 | 77.9 | 2.4 | 0.915 | 0.918 | 0.002 | 225 | 207 | -18 |
| 2004 | 587 | 507 | 0.12 | 51.6 | 47.8 | -3.8 | 0.898 | 0.897 | -0.001 | 198 | 175 | -23 |
| 2005 | 431 | 357 | 0.15 | 83.6 | 81.4 | -2.2 | 0.926 | 0.930 | 0.004 | 180 | 160 | -20 |
| 2006 | 352 | 300 | 0.15 | 22.6 | 22.4 | -0.2 | 0.834 | 0.835 | 0.000 | 89 | 78 | -11 |
| 2007 | 186 | 164 | 0.10 | 61.6 | 55.8 | -5.8 | 0.945 | 0.940 | -0.005 | 89 | 81 | -8 |
| 2008 | 379 | 288 | 0.19 | 50.2 | 47.9 | -2.3 | 0.918 | 0.924 | 0.006 | 119 | 96 | -23 |
| 2009 | 567 | 477 | 0.15 | 48.5 | 45.5 | -3.0 | 0.916 | 0.898 | -0.001 | 133 | 118 | -15 |
| *OL* |  |  |  |  |  |  |  |  |  |  |  |  |
| 2001 | 251 | 235 | 0.11 | 90.4 | 86.6 | -3.8 | 0.945 | 0.948 | 0.002 | 101 | 89 | -12 |
| 2002 | 113 | 98 | 0.11 | 35.2 | 40.2 | 5.0 | 0.906 | 0.903 | -0.003 | 44 | 37 | -7 |
| 2003 | 149 | 117 | 0.16 | 25.2 | 21 | -4.2 | 0.838 | 0.823 | -0.014 | 48 | 37 | -11 |
| 2004 | 199 | 174 | 0.15 | 64 | 60.2 | -3.8 | 0.950 | 0.945 | -0.005 | 81 | 71 | -10 |
| 2005 | 129 | 115 | 0.17 | 72.5 | 80.5 | 8.0 | 0.954 | 0.956 | 0.002 | 63 | 59 | -4 |
| 2006 | 60 | 56 | 0.05 | 98.5 | 74.5 | -24 | 0.949 | 0.945 | -0.005 | 27 | 25 | -2 |
| 2007 | 91 | 67 | 0.33 | 53.9 | 51.1 | -2.8 | 0.953 | 0.950 | -0.003 | 53 | 39 | -14 |
| 2008 | 121 | 114 | 0.18 | 7.1 | 4.1 | -3.0 | 0.678 | 0.650 | -0.028 | 32 | 32 | 0 |
| 2009 | 147 | 115 | 0.20 | 62.3 | 78 | 15.7 | 0.946 | 0.940 | -0.006 | 58 | 45 | -13 |
| *IL* |  |  |  |  |  |  |  |  |  |  |  |  |
| 2001 | 239 | 239 | 0.00 | 38.9 | 38.9 | 0 | 0.935 | 0.935 | 0.000 | 51 | 51 | 0 |
| 2002 | 224 | 223 | 0.01 | 23.1 | 23.6 | 0.5 | 0.902 | 0.904 | 0.002 | 41 | 40 | -1 |
| 2003 | 278 | 273 | 0.02 | 34.8 | 32.5 | -2.3 | 0.905 | 0.904 | -0.001 | 51 | 50 | -1 |
| 2004 | 148 | 148 | 0.00 | 50.2 | 50.2 | 0 | 0.932 | 0.932 | 0.000 | 38 | 38 | 0 |
| 2005 | 77 | 76 | 0.02 | 77 | 79.7 | 2.7 | 0.930 | 0.932 | 0.002 | 27 | 26 | -1 |
| 2006 | 53 | 51 | 0.06 | 31.3 | 32.5 | 1.2 | 0.948 | 0.938 | -0.010 | 19 | 19 | 0 |
| 2007 | 76 | 70 | 0.09 | 58.6 | 53.5 | -5.1 | 0.938 | 0.940 | 0.001 | 25 | 22 | -3 |
| 2008 | 67 | 65 | 0.04 | 37.1 | 39.7 | 2.6 | 0.942 | 0.948 | 0.006 | 20 | 19 | -1 |
| 2009 | 154 | 147 | 0.06 | 39.4 | 47.1 | 7.7 | 0.915 | 0.910 | -0.004 | 35 | 35 | 0 |

Table S5. Effect of addition of genetically divergent subpopulations on genetic summary statistics and $\hat{N}_{b-LDNe}$ for brook trout cohorts from West Brook. Estimates are shown for the WB/OS subpopulation followed by the addition of genetically divergent populations to WB/OS (WB/OS/OL, WB/OS/IL, and WB/OS/OL/IL), where subpopulations added to WB/OS are separated by a ‘/’. Cohorts are defined by the year of emergence. *N*_G_ is the number of individuals genotyped per cohort. $\hat{N}_{fam}$ is the number of estimated full-sib families. $\hat{FE}$ is family evenness, a measure inversely related to variance in the full-sib family distribution of each cohort. $\hat{N}_{b-LDNe}$ (shown with 95% CI) is the effective number of breeders estimated for combined individuals from WB/OS, WB/OS/OL, WB/OS/IL, WB/OS/OL/IL with the program LDNe (assuming random mating). $\hat{N}_{C}$ (shown with 95% CI ) is the number of adults (age-1 and older) estimated from the fall previous to the listed spring, defined cohort. $\hat{N}_{b}/\hat{N}_{C}$is the ratio of both measures.

| Cohort | N_G_ | $\hat{N}_{fam}$ | $\hat{FE}$ | $\hat{N}_{b-LDNe}$ | $\hat{N}_{C}$ | $\hat{N}_{b}/\hat{N}_{C}$ |
| --- | --- | --- | --- | --- | --- | --- |
| *West Brook & Open, small, (WB/OS)* | | | | | | |
| 2001 | 636 | 202 | 0.910 | 68.8 (60.2-78.5) | -- | -- |
| 2002 | 484 | 114 | 0.856 | 25.1 (21.4-29.2) | -- | -- |
| 2003 | 734 | 225 | 0.915 | 75.5 (61.0-92.8) | 846.7 (507.0, 2247.1) | 0.09 |
| 2004 | 587 | 198 | 0.898 | 51.6 (43.5-60.8) | 493.6 (446.7, 564.8) | 0.11 |
| 2005 | 431 | 180 | 0.926 | 83.6 (69.1-101.3) | 338.3 (312.9, 370.0) | 0.25 |
| 2006 | 352 | 89 | 0.834 | 22.6 (17.9-28.0) | 388.2 (354.9, 441.9) | 0.06 |
| 2007 | 186 | 89 | 0.945 | 61.6 (45.8-84.6) | 256.5 (225, 298.4) | 0.24 |
| 2008 | 379 | 119 | 0.918 | 50.2 (41.4-60.6) | 129.0 (114.6, 156.6) | 0.39 |
| 2009 | 567 | 133 | 0.916 | 48.5 (39.4-59.1) | 116.7 (107.2, 136.6) | 0.42 |
| *West Brook, Open, small, and Open, Large (WB/OS/OL)* | | | | | | |
| 2001 | 887 | 278 | 0.926 | 56.7 (49.9, 64.1) | -- | -- |
| 2002 | 597 | 150 | 0.873 | 30.5 (27, 34.2) | -- | -- |
| 2003 | 883 | 255 | 0.913 | 75.6 (64.8, 87.7) | 1075.3 (618.1, 3218.2) | 0.07 |
| 2004 | 786 | 255 | 0.918 | 53.6 (46.9, 60.9) | 704.3 (625.1, 818.7) | 0.08 |
| 2005 | 560 | 225 | 0.935 | 77.8 (68.9, 87.7) | 470.6 (432, 522.2) | 0.17 |
| 2006 | 412 | 110 | 0.848 | 25.6 (21.9, 29.6) | 579.7 (526.7, 666.6) | 0.04 |
| 2007 | 277 | 131 | 0.952 | 69.8 (60, 81.5) | 352.8 (307.4, 413.3) | 0.20 |
| 2008 | 500 | 136 | 0.878 | 31.8 (27, 37) | 220 (193.6, 265.2) | 0.14 |
| 2009 | 714 | 168 | 0.914 | 53.9 (46.9, 61.6) | 208 (182.3, 257.1) | 0.26 |
| *West Brook, Open, small, and Isolated, Large (WB/OS/IL)* | | | | | | |
| 2001 | 875 | 247 | 0.923 | 45.6 (39.1, 52.8) | -- | -- |
| 2002 | 708 | 152 | 0.883 | 27.1 (24.7, 29.6) | -- | -- |
| 2003 | 1012 | 273 | 0.919 | 47.8 (41.2, 55.1) | 1025.9 (600.1, 2852.7) | 0.05 |
| 2004 | 735 | 232 | 0.911 | 48.1 (42.5, 54.3) | 658 (593.1, 752.2) | 0.07 |
| 2005 | 508 | 201 | 0.932 | 67.9 (60, 76.6) | 511.7 (466.7, 569) | 0.13 |
| 2006 | 405 | 102 | 0.859 | 26.9 (23, 31.1) | 498.4 (455.6, 572.1) | 0.05 |
| 2007 | 262 | 113 | 0.949 | 62 (53.2, 72.3) | 319.3 (282.2, 370.4) | 0.19 |
| 2008 | 446 | 138 | 0.927 | 29.8 (26.3, 33.7) | 182.2 (161.1, 222.3) | 0.16 |
| 2009 | 721 | 159 | 0.916 | 51.6 (45.2, 58.5) | 167.9 (151.1, 199.6) | 0.31 |
| *West Brook, Open, small, Open, Large, and Isolated, Large (WB/OS/OL/IL)* | | | | | | |
| 2001 | 1126 | 321 | 0.933 | 45.9 (38.6, 54.1) | -- | -- |
| 2002 | 821 | 188 | 0.891 | 24.3 (21.3, 27.5) | -- | -- |
| 2003 | 1161 | 301 | 0.918 | 44.3 (38, 51.4) | 1254.5 (711.2, 3823.8) | 0.04 |
| 2004 | 934 | 287 | 0.926 | 45.3 (39.4, 51.8) | 868.7 (771.6, 1006) | 0.05 |
| 2005 | 637 | 246 | 0.939 | 72.5 (64.2, 81.6) | 644 (585.8, 721.1) | 0.11 |
| 2006 | 465 | 123 | 0.867 | 25.1 (21.4, 29.1) | 689.9 (627.4, 796.8) | 0.04 |
| 2007 | 353 | 153 | 0.953 | 55.9 (48, 65.2) | 415.6 (364.5, 485.3) | 0.13 |
| 2008 | 567 | 155 | 0.891 | 46.2 (40.1, 53) | 273.1 (240.1, 331) | 0.17 |
| 2009 | 868 | 192 | 0.926 | 47.7 (41.5, 54.4) | 259.2 (226.2, 320.1) | 0.18 |

Fig. S1

Fig. S2

Fig. S3

Fig. S4

Fig. S5
